# Transfer entropy as a variable selection methodology of cryptocurrencies in the framework of a high dimensional predictive model

**DOI:** 10.1371/journal.pone.0227269

**Published:** 2020-01-02

**Authors:** Andrés García-Medina, Graciela González Farías

**Affiliations:** 1 Consejo Nacional de Ciencia y Tecnología, Av. Insurgentes Sur 1582, Col. Crédito Constructor 03940, Ciudad de México, México; 2 Unidad Monterrey, Centro de Investigación en Matemáticas, A.C. Av. Alianza Centro 502, PIIT 66628, Apodaca, Nuevo Leon, Mexico; 3 Probability and Statistics, Centro de Investigación en Matemáticas, A.C. Jalisco S/N, Col. Valenciana 36240, Guanajuato, Mexico; University of Almeria, SPAIN

## Abstract

We determine the number of statistically significant factors in a high dimensional predictive model of cryptocurrencies using a random matrix test. The applied predictive model is of the reduced rank regression (RRR) type; in particular, we choose a flavor that can be regarded as canonical correlation analysis (CCA). A variable selection of hourly cryptocurrencies is performed using the Symbolic estimation of Transfer Entropy (STE) measure from information theory. In simulated studies, STE shows better performance compared to the Granger causality approach when considering a nonlinear system and a linear system with many drivers. In the application to cryptocurrencies, the directed graph associated to the variable selection shows a robust pattern of predictor and response clusters, where the community detection was contrasted with the modularity approach. Also, the centralities of the network discriminate between the two main types of cryptocurrencies, i.e., coins and tokens. On the factor determination of the predictive model, the result supports retaining more factors contrary to the usual visual inspection, with the additional advantage that the subjective element is avoided. In particular, it is observed that the dynamic behavior of the number of factors is moderately anticorrelated with the dynamics of the constructed composite index of predictor and response cryptocurrencies. This finding opens up new insights for anticipating possible declines in cryptocurrency prices on exchanges. Furthermore, our study suggests the existence of specific-predictor and specific-response factors, where only a small number of currencies are predominant.

## Introduction

In econometrics, it is of fundamental interest to determine the proper number of components in a multivariate model because this allows for the attribution of explanatory meaning to each factor based on economic theory. Traditionally, a visual inspection approach has been a standard methodology in factor analysis and principal component analysis (PCA) since the seminal publication [1]. There, a technique known as the scree test, whereby the eigenvalues associated with the covariance matrix are ordered from largest to smallest and plotted as a downward curve, was proposed. According to the test, we must look for an *elbow* in the curve and retain the factors associated with the eigenvalues to the left of that point. Of course, this methodology has the disadvantage of being highly subjective, and different researchers can choose different numbers of factors. Another usual approach originates from purely nonparametric statistics and relies on the cross-validation technique [2]. Methods of this type are computationally demanding and become impractical if the number of variables increases at the same rate as the number of observations in the model.

A more recent approach using a combination of parametric and nonparametric assumptions has been proposed in [3]. The researchers include as significant factors quantities for which the mean eigenvalue distribution under a bootstrapping resampling is larger than one. The latter criterion is attributed to Kaiser [4] but was proposed analytically by Guttman [5]. This method is also biased if the number of variables increases with that of observations. In this case, it is better to use a modified version of the Kaiser approach [6] to perform analyses in this regime known as high-dimensional statistics. In high-dimensional factor analysis, there are relevant proposals for determining the number of factors that originate in random matrix theory [7, 8]. Even in the related estimation method based on ratio tests of eigenvalues [9], some conclusions are consequences of the results for random matrices. In the standard use of random matrices, the Tracy-Widom distribution [10] plays a crucial role in testing the sample eigenvalue distribution. Additionally, more sophisticated approaches use the joint Tracy-Widom distribution [8] to determine the number of factors, and further elegant versions use free matrices and noncommutative probability [11, 12]. Nevertheless, all the novel approaches in the high-dimensional regime are mainly focused on PCA and factor analysis, while other multivariate models such as the canonical correlation analysis (CCA) have drawn less attention.

In this study, we are interested in a model of the latter type, where CCA is presented as a particular case of the reduced-rank regression model. In this case, Johnstone [13] proved that the Tracy-Widom distribution could be used to determine the number of significant factors after appropriate transformations of the greatest root distribution involved in the Union Intersection Test (UIT). Based on this technique, the idea is to test the null hypothesis *H*_0_: **Σ**_*xy*_ = I, where **Σ**_*xy*_ represents the covariance matrix between Gaussian datasets **X** and **Y**. In some sense, not imposing a structure simplifies the analysis. However, it is important to keep in mind that we will follow a parametric approach, and some restrictions are imposed accordingly. The relevant assumption here is that the data must follow a normal distribution, which usually is untrue for financial time series in the high-frequency domain [14]. Considering this problem, Burda et al. [15, 16] have derived a heavy-tail limit distributions of eigenvalues based on the framework of random matrices. The researchers determined that in the limit case of Levy processes, the distribution of eigenvalues of the sample correlation matrix does not have a bounded support. Hence, no limit distribution of the largest eigenvalues exists as an equivalent to the Tracy-Widom distribution. Therefore, the results obtained in this study can be thought as an upper bound for the maximum number of significant factors, and the true number can be less if the relevant time series is heavy-tailed. But as an intermediate case, if the density of the matrix entries behaves like |*x*|^−*μ*^, then there exist physical arguments to support a phase transition from Tracy–Widom to Poisson at *μ* = 4 [17]. Then, this enable to use the Tracy-Widom statistics to financial time series with moderate heavy-tail behavior.

Thus, our intention is to implement the Tracy-Widom test to determine the number of significant factors in a predictor-response set of cryptocurrencies modeled by CCA. These new financial instruments are based on blockchain [18] technology, where a coin is defined as a chain of digital signatures. Each owner transfers the coin to the next owner by digitally signing a hash of the previous transaction and the public key of the next owner and adding these to the end of the coin. The easy access to this new financial instrument through more than 17000 exchanges with low transaction fees, more than 2000 virtual currencies worldwide and a traded volume of nearly 60 billion dollars has made it a very attractive investment instrument for the general population [19].

Interestingly, several studies have shown evidence that such instruments are inefficient in the meaning of the efficient market hypothesis [20]. Consequently, predictive strategies can be applied to earn profits from trading on the relevant virtual exchange platforms. To mention some examples, in [21], Bitcoin was studied over the historical period of 2010-2016 with a battery of robust tests, and the study concluded that Bitcoin was transitioning from an inefficient market to an efficient one. Another study [22] has exploited this inefficiency based on a machine learning framework and searched for abnormal profits using a representative set of cryptocurrencies traded on various exchanges during 2015-2018. Additionally, the above study presented several insights for the prediction of the short-term evolution of the cryptocurrency market. Another study [23] in this direction observed persistence in four major cryptocurrencies, i.e., a positive correlation between a cryptocurrency’s past and future values, and similarly concluded that unusual profits could be earned by trading in these markets. Hence, given the above studies, it does not seem unreasonable to study the statistical determination of the number of factors in a multivariate predictive model, e.g., using CCA.

Similarly, attempts have been made to characterize the collective behavior of cryptocurrencies; one example is [24]. There, it was shown that a large dataset of cryptocurrencies at the daily frequency deviated from the universal results of Marchenko-Pastur [25]. Additionally, the study stated that the spanning tree structure was stable over time. Furthermore, the power-law behavior of Bitcoin was analyzed in [26] over a long period of time and at various frequencies from one minute to one day. The researchers concluded that Bitcoin exhibited heavy tails in the range of 2 < *α* < 2.5 across multiple coin exchanges. Their findings supported the use of standard financial methods because of the finite variance implications of results. Nonetheless, we are interested in remaining within the domain of the parametric approach of random matrices since the computational cost is minimal and only a few assumptions are required.

On the other hand, a crucial step in any predictive model is the variable selection procedure used to define the predictor and response variables. In most cases, this is done based on the accumulated research experience or according to the consensus of experts in the field. However, cryptocurrencies are a new financial instrument for which there is a limited amount of previous experience in making this selection. Thereby, this study proposes using the transfer entropy measure to solve the variable selection problem. Transfer entropy is a dynamic and nonsymmetric measure that was initially developed by Schreiber [27] and is based on the concept of Shannon entropy [28]. This measure was designed to determine the directionality of transfer information between two processes by detecting the asymmetry in their interactions [29]. Transfer entropy has been used to solve numerous problems. It has been useful in the study of the neuronal cortex of the brain [30], statistical physics [31], and dynamic systems [32], and was given a thermodynamic interpretation in [33]. In applications to econometrics, transfer entropy can be regarded as a nonlinear generalization of the Granger causality test [34]. In this field, effective transfer entropy [35] has been proposed for dealing with finite sample effects. Nevertheless, it does not have an empirical limit distribution that can be used as a comparison. Instead, effective transfer entropy is based in resampling and the use of surrogate data. The study of Sandoval [36] uses this approach to study the contagion of institutions in times of crisis. The cited study identifies the companies most vulnerable to contagion and dependent on failing economies. A more recent study in this direction is [37], where transfer entropy is estimated by discretizing the return time series into positive and negative unit values. The researchers used stock market and real estate data to construct an indicator used to measure systematic risk to predict future market volatility.

In the above applications, the estimation method is based on binning. Our intention is to use the symbolic approach, where the time series can be thought of as embedded in a dynamic system. This avoids fine-tuning of parameters, which usually limits the use of transfer entropy to field applications. Symbolic transfer entropy is a robust and computationally fast method of quantifying the dominant direction of information flow between time series [38]. Furthermore, in [39], the multivariate version of symbolic transfer entropy has been tested, and it has been shown that it can be applicable to nonstationary time series in mean and variance and is even unaffected by the existence of outliers and vector autoregressive filtering. Moreover, another advantage of using the symbolic approach is that under some circumstances, there exists a null hypothesis regarding the distribution that can be used to measure the absence of a direct flow of information. This makes the results more robust and simpler to compute.

To end this section, let us point out that, in this study, our concerns are focused on the proper determination of the number of components and variable selection beyond the predictive precision of the CCA model. Thus, the aim of this study is twofold. First, it is to provide tools related to variable selection and the number of statistically significant factors in a multiresponse forecast obtained with CCA that describes an apparently unrelated mathematical apparatus. Second, it is to explore the methodology’s application to the new cryptocurrency instruments. However, the proposed approach is general, and this framework can be applied to the analysis of any dataset of interest.

In the next section, the preprocessing of the dataset of cryptocurrencies is described. Next, in the section on variable selection, the transfer entropy measure is used to discriminate between the set of predictor and response variables, and the results for cryptocurrency-related variables are presented. The regression framework section introduces the general regression model that serves as the setting for the studied model. Then, in the section on the number of factors, random matrix theory is used to select the appropriate number of factors in the presented multiresponse regression model considered at a high-dimensional setting. The mathematical relation of results in high-dimensional statistics with the reduced-rank selection problem for the particular case of CCA is also described there. Next, the consequences of the methodology are explored by considering the set of predictor-response cryptocurrencies. Finally, in the concluding section, the main findings are summarized, and future research directions are proposed.

## Data

A sample of *p* = 100 cryptocurrencies is obtained using the API of CoinMarketCap [19] for the period from May 23 to November 27, 2018, at an hourly frequency, resulting in a total of *n* = 4533 observations (see S1 File and S1 Table).

We transform exchange rates in dollars *P*_*k*_(*t*) to returns *R*_*k*_(*t*) for every cryptocurrency (*k* = 1, …, *p*) and moment of time (*t* = 1, …, *n*)

$$\begin{array}{c} R_{k}\left(t\right)=\frac{Z_{k}\left(t+\Delta t\right)-Z_{k}\left(t\right)}{Z_{k}\left(t\right)}, \end{array}$$ (1)

and analyze the standardized returns *r*_*k*_ = (*R*_*k*_ − *μ*_*k*_)/*σ*_*k*_ (*k* = 1, …, *p*), where *σ*_*k*_ is the standard deviation of *R*_*k*_, and *μ*_*k*_ denotes the average over time for the studied period. Due to this transformation, the augmented Dickey-Fuller test [40] confirms that the relevant time series are stationary, with a *p-value* of less than 0.01 for all *r*_*k*_ (*k* = 1, …, *p*) considered.

## Variable selection

One of the first problems encountered when trying to establish a predictive model is variable selection. In the econometric approach, the economic theory usually dictates which variables must be treated as predictors and as responses. However, cryptocurrencies are a new financial instrument with not many economic models available. Hence, we follow an information-theoretic approach to solve the variable selection problem.

In 2000, T. Schreiber introduced a quantity called transfer entropy (TE) in the context of information theory, with the purpose of measuring the information flow from one process to another in a nonsymmetrical way. Let *x*_*i*_ = *x*(*i*) and *y*_*i*_ = *y*(*i*), *i* = 1, …, *N*; denote sequences of observations of systems *X* and *Y*. TE is defined as [27]

$$\begin{array}{c} T_{Y\to X}\left(k,l\right)=\sum_{i,j}p\left(x_{t+1},x_{t}^{\left(k\right)},y_{t}^{\left(l\right)}\right)\log\frac{p(x_{t+1}|x_{t}^{\left(k\right)},y_{t}^{\left(l\right)})}{p(x_{t+1}|x_{t}^{\left(k\right)})}, \end{array}$$ (2)

The idea behind TE is to incorporate time dependence by relating previous samples *x*_*i*_ and *y*_*i*_ to predict the next value *x*_*i*+1_ and quantify the deviation from the generalized Markov property, *p*(*x*_*i*+1_|*x*_*i*_, *y*_*i*_) = *p*(*x*_*i*+1_|*x*_*i*_), where *p* denotes the transition probability density. If there is no deviation from the generalized Markov property, *Y* has no influence on *X*. TE, formulated as the Kullback-Leibler entropy [41] between *p*(*x*_*i*+1_|*x*_*i*_, *y*_*i*_) and *p*(*x*_*i*+1_|*x*_*i*_), quantifies the incorrectness of this assumption and is explicitly nonsymmetric with respect to the exchange of *x*_*i*_ and *y*_*i*_.

An interesting property of TE is that under some conditions it can be regarded as a nonlinear generalization of Granger causality. In econometrics, Granger causality plays an important role in parameter estimation of a vector autoregressive (VAR) model. Consider the jointly stationary stochastic processes *X*_*t*_, *Y*_*t*_. Let $F(x_{t}|x_{t-1}^{\left(k\right)},y_{t-1}^{\left(l\right)})$ denote the distribution function of the target variable *X*, conditional on the joint (*k*, *l*) history $X_{t-1}^{\left(k\right)},Y_{t-1}^{\left(l\right)}$. Then, variable *Y* is said to be Granger-cause variable *X* (with lags *k*, *l*) if and only if [42, 44]

$$\begin{array}{c} F(x_{t}|x_{t-1}^{\left(k\right)},y_{t-1}^{\left(l\right)})\neq F(x_{t}|x_{t-1}^{\left(k\right)}). \end{array}$$ (3)

Thereby, it is said that *Y* Granger-causes *X* if and only if *X* is not independent of the history of *Y*.

There exists a series of results [31, 34, 45] that state an exact equivalence between the Granger causality and TE statistics for various approaches and assumptions on the data generating processes, which make it possible to construct TE as a nonparametric test of pure Granger causality. This connection can be regarded as a bridge between causal inference of data under autoregressive models and the information-theoretic approach. Before proceeding, we want to emphasize that for highly nonlinear and non-Gaussian data, as is the case for many financial instruments, it is better to approach causality by using the TE information method instead of the traditional Granger causality test [44].

There are several techniques for estimating TE from observed data in order to apply it to real-world data problems. However, most of them require a large amount of data, and consequently, their results are commonly biased due to small-sample effects, which limits the use of TE in practical data applications. To avoid this problem, we use the robust and computationally fast technique of symbolization [38] to estimate TE. Symbolic transfer entropy (STE) has been introduced within the concept of permutation entropy [46]. Following [38, 46], symbols are defined by reordering the amplitude values of time series *x*_*i*_ and *y*_*i*_. Thus, for a given *i*, *m* arbitrary amplitude values, the elements of the delay vector

$$\begin{array}{c} \{x(i),x(i+l),\cdots ,x(i+(m-1)l)\}, \end{array}$$ (4)

are arranged in ascending order

$$\begin{array}{c} \{x\left(i+\left(k_{i1}-1\right)l\right)\leq x\left(i+\left(k_{i2}-1\right)l\right)\leq \cdots \leq x\left(i+\left(k_{im}-1\right)l\right)\}, \end{array}$$ (5)

where *l* denotes the time delay, i.e., the time between successive observations of *x*(*i*); and *m* represents the embedding dimension, which is interpreted in the time series context as the total number of past data points in the delay vector. A symbol is thus defined as ${\hat{x}}_{i}=\left(k_{i1},k_{i2},\ldots ,k_{im}\right)$, and the relative frequency of symbols is used to estimate the joint and conditional probabilities of the sequence of permutation indices. One of the advantages of STE is the delay vector formed by time series segments can be compared in terms of rank. Thus, similar data patterns can be found regardless of their magnitude levels, extending its validity to non-stationary systems [39].

To provide an example of this procedure, let us consider the time series {1, 2, 3, 6, 5, 4} to estimate the related Shannon entropy [28] measure for *l* = 1, *m* = 2. First, we need to organize the five pairs of adjacent figures according to their relative values. As a result, three pairs are observed to satisfy the relation *x*_*t*_ < *x*_*t*+1_ characterized by permutation {01}, and two pairs for which *x*_*t*_ > *x*_*t*+1_ represent permutation {10}. Then, the Shannon entropy for *l* = 1, *m* = 2 is given by

$$\begin{array}{c} H(2)=-(3/5)\log(3/5)-(2/5)\log(2/5)\approx 0.971. \end{array}$$ (6)

Let us now return to the original problem of TE estimation. Given symbol sequences $\left\{{\hat{x}}_{i}\right\}$ and $\left\{{\hat{y}}_{i}\right\}$, STE is mathematically defined as [38]

$$\begin{array}{c} T_{Y\to X}^{S}=\sum_{i,j}p\left({\hat{x}}_{i+\delta },{\hat{x}}_{i},{\hat{y}}_{i}\right)\log\frac{p({\hat{x}}_{i+\delta }|{\hat{x}}_{i},{\hat{y}}_{i})}{p({\hat{x}}_{i+\delta }|{\hat{x}}_{i})}, \end{array}$$ (7)

where the sum is over all symbols, and *δ* denotes a time step. The logarithm is with base 2; thus, $T_{Y\to X}^{S}$ is in bits.

The question at this point is whether a given empirical measurement of STE is statistically different from 0 and represents sufficient evidence of a direct relationship between the variables. It is possible to construct a null hypothesis *H*_0_ that there is no such relationship, but it is necessary to know what the distribution of an empirical measurement would look like if *H*_0_ were true and then evaluate a *p-value* for sampling the actual measurement from the distribution. If the test fails, we will accept the alternate hypothesis that there exists a direct relationship.

For discrete *X* and *Y*, it is known that if *H*_0_ is true, then $T_{Y^{s}\to X}^{S}\overset{d}{\to }\chi^{2}\left(d\right)/\left(2N\log2\right)$, where the number of degrees of freedom *d* is the difference between the number of parameters in the full and null models [31]. *Y*_*s*_ represents surrogate variables for *Y* generated under *H*_0_, which have the same statistical properties as does *Y*, but any potential correlation with *X* is destroyed. As a consequence, surrogates of the distribution $T_{Y^{s}\to X}^{S}$ must preserve $p({\hat{x}}_{i+\delta }|{\hat{x}}_{i})$ but not $p({\hat{x}}_{i+\delta }|{\hat{x}}_{i},{\hat{y}}_{i})$ [47].

In order to evaluate the advantage of STE over the Granger causality approach let us consider the following simulated systems:

1. *A three-variable system, with nonlinear drivers*
  $$\begin{array}{c} \begin{array}{cc} x_{1}\left(t\right) & =0.7x_{1}\left(t-1\right)+\varepsilon_{1}\left(t\right)\\ x_{2}\left(t\right) & =0.3x_{2}\left(t-1\right)+0.5x_{2}\left(t-2\right)x_{1}\left(t-1\right)+\varepsilon_{2}\left(t\right)\\ x_{3}\left(t\right) & =0.3x_{3}\left(t-1\right)+0.5x_{3}\left(t-2\right)x_{1}\left(t-1\right)+\varepsilon_{3}\left(t\right), \end{array} \end{array}$$ (8)
  where *ε*_*i*_ ∼ *N*(0, 1).
  The term product of the variables in the second and third equation causes the variables *x*_2_ and *x*_3_ to have marginal distributions with long tails [39]. Therefore this model characterizes the heavy-tail behavior of financial time series.
2. *A ten-variable system with overlapping linear drivers separated in two blocks*
  $$\begin{array}{c} x_{i}\left(t\right)=\sum_{j}^{10}A_{ij}x_{j}\left(t-1\right)+\varepsilon_{i}\left(t\right),\;i=1,\cdots ,10; \end{array}$$ (9)
  where *ε*_*i*_ ∼ *N*(0, 1), and the interaction matrix *A* has the form
  $$\begin{array}{c} A=(\begin{array}{cccccccccc} a & b & c & 0 & 0 & 0 & 0 & 0 & 0 & 0\\ 0 & a & b & c & 0 & 0 & 0 & 0 & 0 & 0\\ 0 & 0 & a & b & c & 0 & 0 & 0 & 0 & 0\\ 0 & 0 & 0 & a & b & 0 & 0 & 0 & 0 & 0\\ 0 & 0 & 0 & 0 & a & 0 & 0 & 0 & 0 & 0\\ 0 & 0 & 0 & 0 & 0 & a & b & c & 0 & 0\\ 0 & 0 & 0 & 0 & 0 & 0 & a & b & c & 0\\ 0 & 0 & 0 & 0 & 0 & 0 & 0 & a & b & c\\ 0 & 0 & 0 & 0 & 0 & 0 & 0 & 0 & a & b\\ 0 & 0 & 0 & 0 & 0 & 0 & 0 & 0 & 0 & a\; \end{array}) \end{array}$$ (10)
  where we set *a* = 0.9, *b* = 0.7, and *c* = 0.3.

In the simulations of each system, a time series length of dimension *T* = 2048 is considered after removed the transitory effect of 256 steps. For STE the time delay is set to *l* = 1 as usual practice, and the embedding dimension is set to *m* = 2 for both systems. The embedding specification is because the maximum lag is two for *system 1*, as well as *m* must be bigger than one by definition. Likewise, the order *P* of the VAR model associated to the Granger causality test is set to *P* = 2 for *system 1*, and *P* = 1 for *system 2* in accordance with the lag order of each model. As a criterion of a causal relation, the null hypothesis is rejected at the level of *p-value* = 0.01. In the Granger approach has been considered the F-distribution and the chi-square distribution to test the coefficients of the causal time series. Thus, the null of zero value coefficients must be rejected in both tests in order to consider a true causal relation, and the F-value is used as the quantity to compare the magnitude of the causal relationships. Further, the visual representation is done via a directed graph *G* = (*V*, *E*), where the nodes *V* represent the involved variables, and the direction of the edges *V* represent the causal relations given by the significative STE or F values, for the transfer entropy and Granger causality approach, respectively.

**Fig 1:**
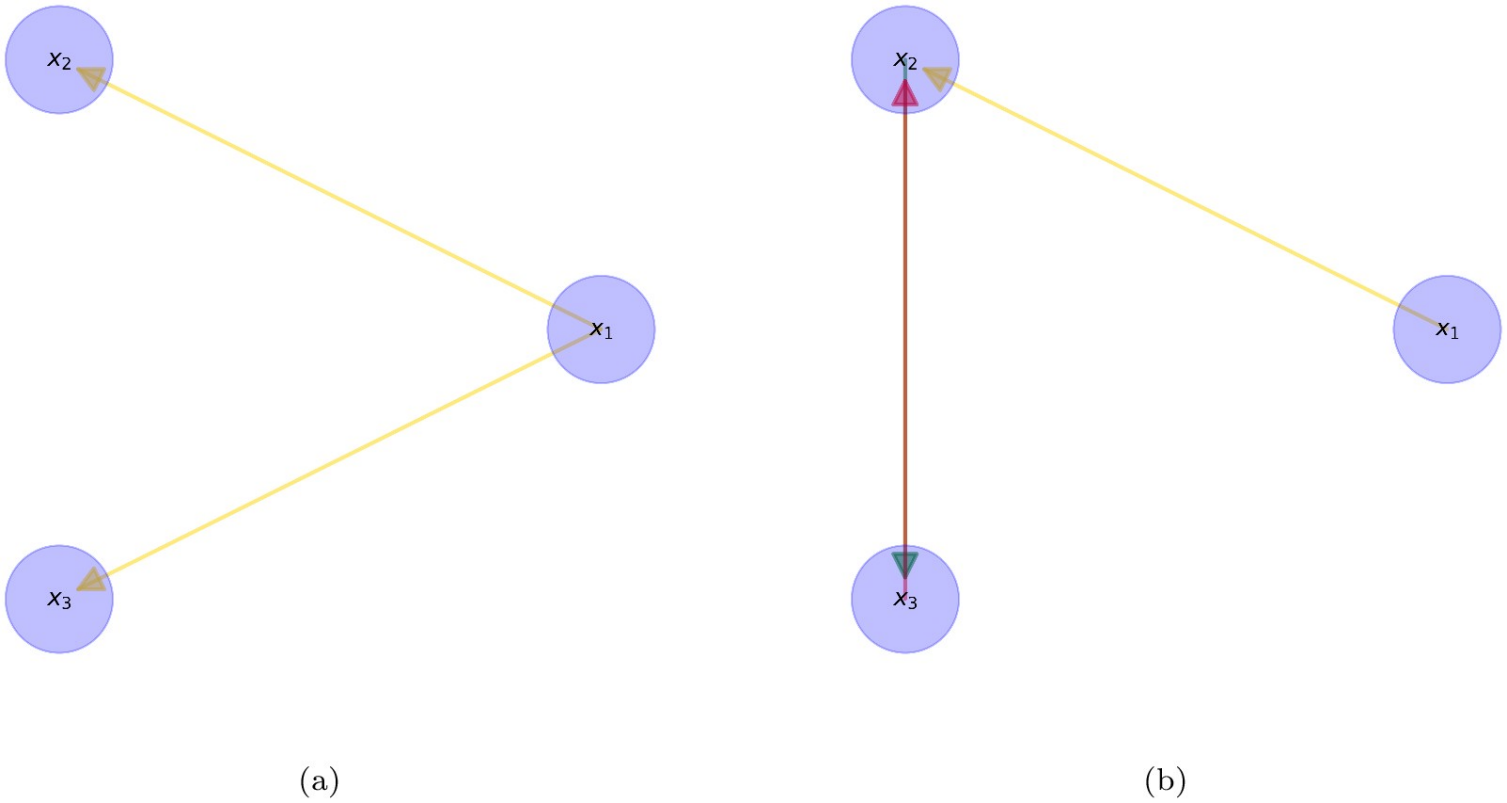
System 1. (a) Represents the STE detected interactions (b) Represents the Granger causality detected interactions.

In *system 1* it is modeling a system with nonlinear drivers *x*_1_ → *x*_2_ and *x*_1_ → *x*_3_, where the variables *x*_2_ and *x*_3_ comes from a distribution with long-tails. In Fig 1 it is shown the associated directed graph for (a) STE and (b) Granger causality measures, respectively. We can notice STE correctly detects the nonlinear direct causality, whereas Granger causality is not able to describe the two nonlinear interactions, but on the contrary indicates two spurious causal effects *x*_2_ → *x*_3_ and *x*_3_ → *x*_2_.

**Fig 2:**
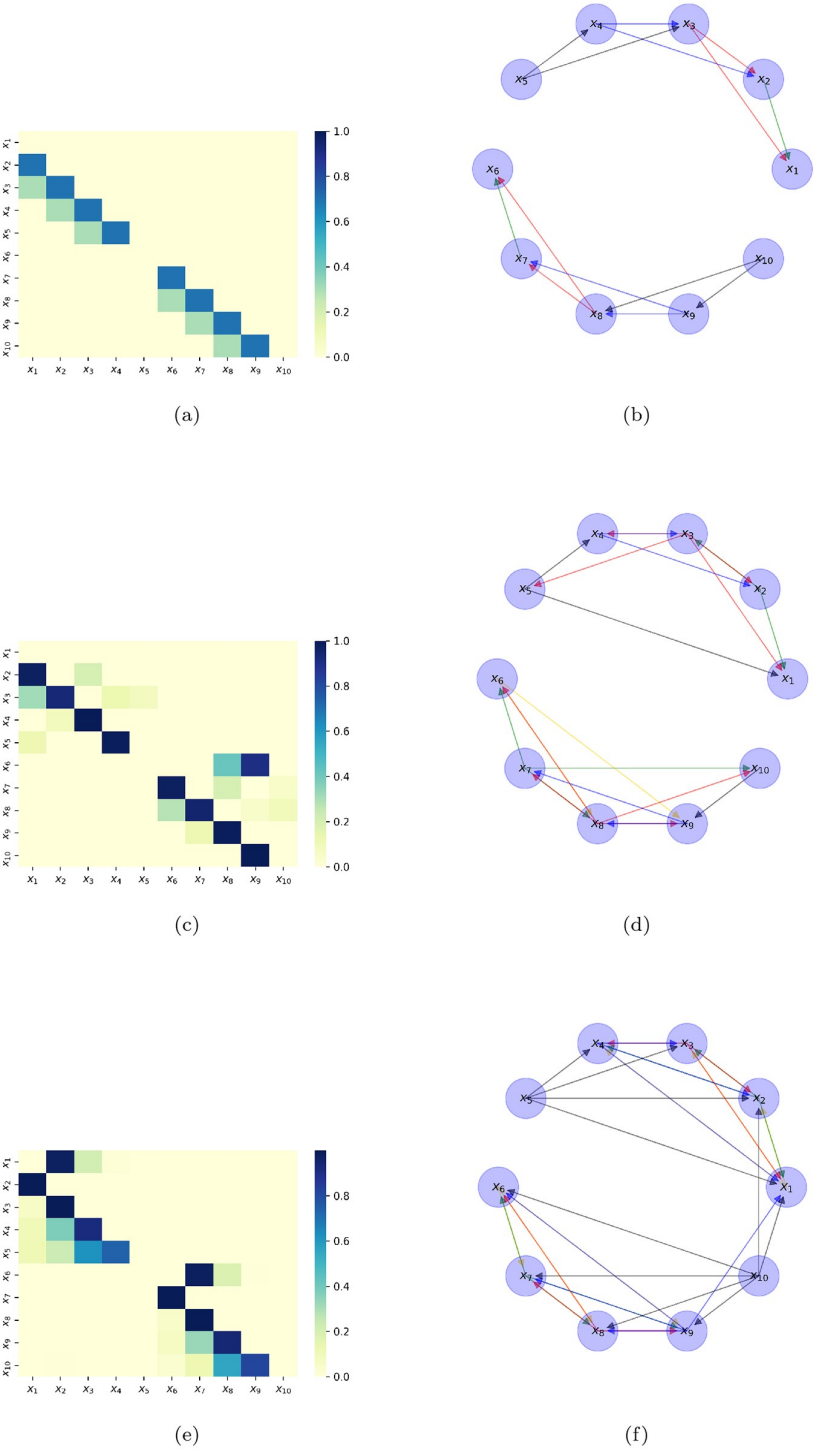
System 2. (a) Heatmap of the interaction matrix A. (b) Directed graph of the interaction matrix A. (c) Heatmap of the STE detected interactions. (d) Directed graph of the STE detected interactions. (e) Heatmap of the Granger causality detected interactions. (f) Directed graph of the Granger causality detected interactions.

In *system 2*, it is modeling a system where each variable is linearly driver by at most two variables, and the interactions as a whole are separated into two groups. The linear interaction matrix *A* is shown in Fig 2a as a heatmap, and in Fig 2b as a directed graph. In the same way, Fig 2(c), 2(d), 2(e) and 2(f) shown the detected causal relations by STE and Granger causality, respectively. We can notice STE only detects causal effects within the cluster, while Granger causality on the contrary also detects spurious interactions between clusters. Nevertheless both approaches detect spurious relations within clusters, the number of false-positive interactions is less for STE than for Granger causality, with 10 and 21, respectively. On the other hand, the number of false-negatives is two for STE, whereas Granger causality is zero. In Fig 2c and 2e is shown the scaled magnitude of STE and Granger Causality in values between zero and one, in this scaled representation, the magnitude of the false-positives interactions in the Granger approach is 33% more than in the STE approach.

In the last models, we have tested linear and nonlinear causal interactions. In the nonlinear model, the Granger causality approach does not was able to detect the interactions and even indicates two more spurious drivers, while STE correctly describes the system. In the linear model, both approaches detects spurious drivers, but the number and magnitude were bigger under the Granger measure. Also, it does not discriminate against the cluster structure of the system. Despite the fact we have tested causalities in bivariate form, STE can capture the main characteristics of the system, which allows us to rely on its use for the applications as the study we are interested in below.

### Application to cryptocurrency data

To present our results in the context of a predictive model, let us refer to variable *x*(*t*) as the predictor and variable *y*(*t*) as the response. Thus, we estimate STE for the combination of pairs {*X*_*a*_(*t*), *Y*_*b*_(*t* + Δ*t*)}, where *a*, *b* = 1, …, *p* (= 100); *t* = 0, …, *n* − Δ*t*, and Δ*t* is a time lag added to consider predictive scenarios. The STE estimation needs the specification of the time delay *l* and the embedding dimension *m* as has been explained above. In this study *l* is fixed and *m* is varied as usual practice to simplify the state-space reconstruction [43]. The results for time delay *l* = 1 and *p-value* = 0.10 are given in Table 1 for various values of lag time Δ*t* and embedding dimension *m*. The third column shows the total sum of $T_{X_{a}^{s}\to Y_{b}}^{S}$ for all possible combinations of indices *a*, *b*, as long as a direct relationship exists under *H*_0_. The fourth column shows the number of relations that are preserved. We observe a peak in the number of preserved relations at Δ*t* = 1 and *m* = 2, 3, where the number of relations exceeds 7000 out of 10000 possible relations. Even though the maximum is reached at *m* = 2, we chose the case *m* = 3 following the criterion of obtaining at the same time the maximum of the total sum of information flow (118.1084 bits).

**Table 1:** STE results.

Δ*t*	*m*	$\sum_{ab}T_{ab}^{S}$	#{$T_{ab}^{S}>0$}
0	2	7.7484	4221
0	3	97.7024	6345
0	4	241.6957	736
**1**	**2**	**19.677**	**7756**
**1**	**3**	**118.1083**	**7067**
1	4	351.52	1069
2	2	1.3937	1289
2	3	68.196	4701
2	4	442.0707	1342
3	2	1.3346	1240
3	3	13.8508	1070
3	4	333.1614	1013

STE of cryptocurrency return time series for each of lag times Δ*t* = 0, 1, 2, 3 for the pair of predictor-response variables *X*, *Y* and embedding dimension *m* = 2, 3, 4. The third column shows the total amount of direct information at the *p-value* of 0.10, while the fourth column shows the corresponding number of preserved relations at the same level of statistical significance.

**Fig 3:**
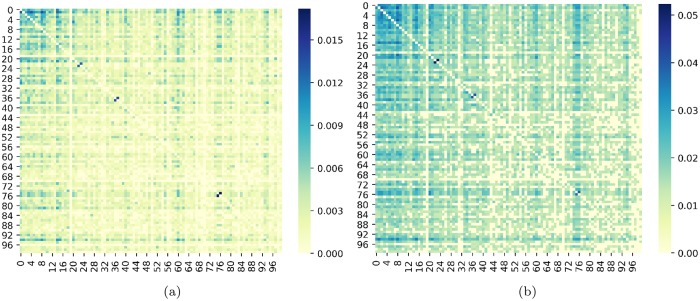
Heat map of STE. (a) *m* = 2. (b) *m* = 3. The color intensity represents the magnitude of STE.

Moreover, we show in Fig 3a and 3b the heat map of STE results for *m* = 2 and *m* = 3, respectively. It is observed that STE has higher values in Fig 3b than in Fig 3a in general. Further, some structure can be noticed in the upper left section of Fig 3a, which is sharper in Fig 3b. This upper left section corresponds to cryptocurrencies with the highest capitalization due to the way we order them. Therefore, it is natural to have the highest values of information flow in that sector.

A convenient procedure for measuring the net flow of information between processes *X* and *Y* is by using the normalized directionality index (NDI), given by [44]

$$\begin{array}{c} d\left(X,Y\right)=\frac{STE_{X\to Y}-STE_{Y\to X}}{STE_{X\to Y}+STE_{Y\to X}}\in \left[-1,1\right]. \end{array}$$ (11)

This quantity standardizes *STE* values to be between −1 and 1, so *d*(*X*, *Y*) is maximized if one of the *STE* values is zero and minimized if they are equal. This index is not normalized in the statistical sense but resembles a measure of divergence or market leverage and, beyond that, is very useful in comparing measures across different systems or financial sectors. The form of Eq (11) gives importance to the relative information flows between *X* and *Y* enabling compare different relations in the same scale. This feature help us to consider as a drivers of *Y*, the *X*’s variables for which *d*(*X*, *Y*) > 0. In this way we set the prefered direction of information flow and avoid the ambiguity of having both directions with different weights, which help to compute the variable selection in straightforward manner by simple concepts of directed graph.

**Fig 4:**
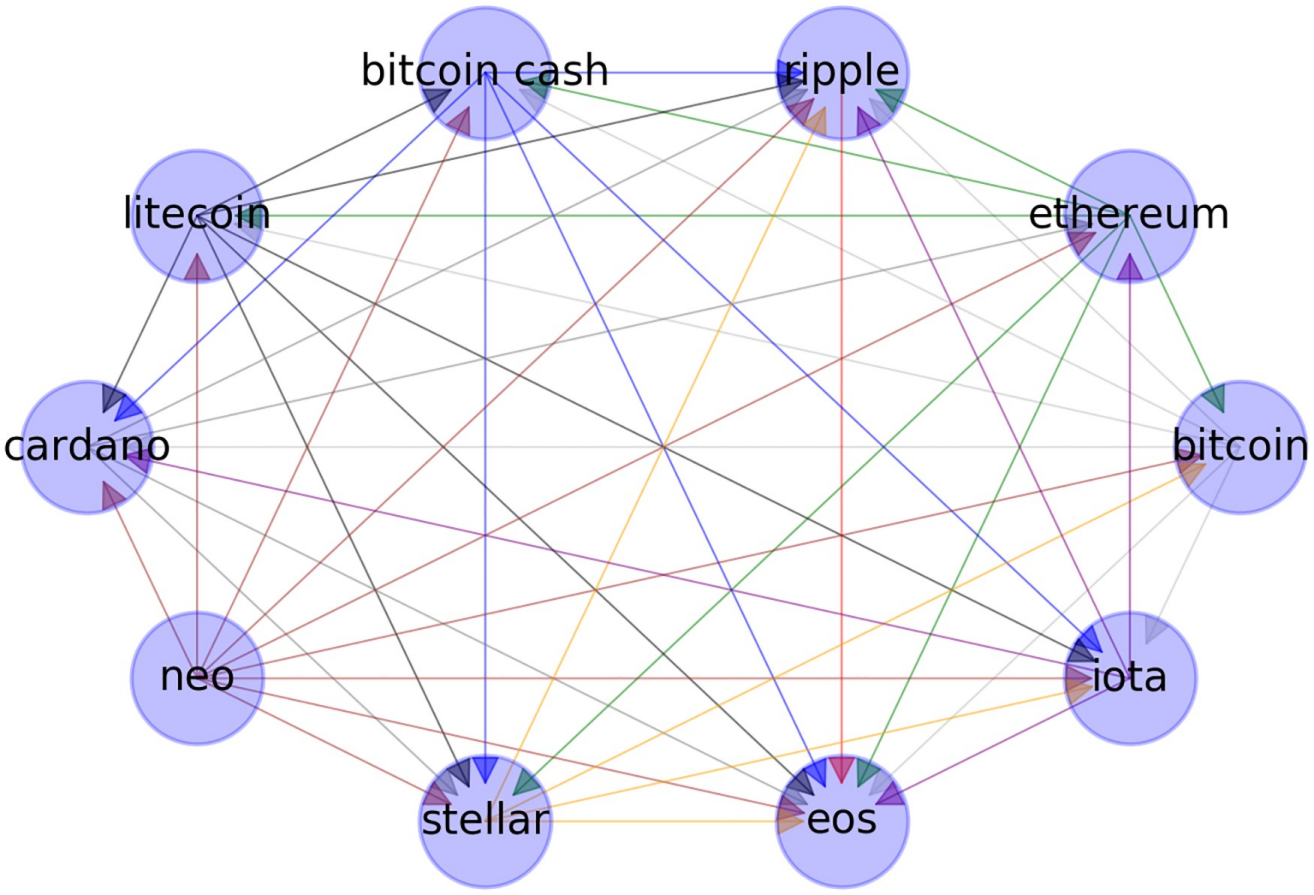
NDI subgraph. The arrow direction represents the direction of the information flow.

We apply NDI to our previous results for Δ*t* = 1 and *m* = 3. Again, for a better visualization, the obtained values are first converted to a directed graph *G* = (*V*, *E*), where the nodes *V* represent cryptocurrencies, and the edges *E* now represent the positive values of *d*(*X*, *Y*) resulting from applying NDI. Fig 4 shows as an example a directed subgraph for the first 10 cryptocurrencies in the order of capitalization with its corresponding edges given by the NDI measure. In this figure, the arrow direction indicates the information flows from one variable to another. We observe, e.g., that the *eos* coin only receives information from the other cryptocurrencies under the NDI measure, whereas the *ripple* sends and receives information from the members of the subnet.

To discriminate the predictor variables from the response variables, several basic concepts of graph theory are used. The node out-degree *k*^*out*^ is the number of edges pointing out from the node, while the node in-degree *k*^*in*^ is the number of edges pointing towards the node. We use these concepts to select the sets of predictor-response variables by the proposed heuristic selection rule:

$$\begin{array}{c} \begin{array}{c} V_{i}\in \left\{\text{response}\;\text{variables}\right\}\;\text{if}\;k_{i}^{in}\geq k_{i}^{out},\\ V_{i}\in \left\{\text{predictor}\;\text{variables}\right\}\;\text{if}\;k_{i}^{in}<k_{i}^{out}, \end{array} \end{array}$$ (12)

for *i* = 1, …, *p*. The results of applying this procedure are shown in Table 2 for the first 10 response and predictor variables (see S2 Table for the entire list). In general, we found 49 predictor variables and 51 response variables in our set of *p* = 100 times series of cryptocurrency returns.

**Table 2:** Predictor and response variables.

i	Predictor (49)	Response (51)
1	ethereum	bitcoin
2	neo	ripple
3	dash	bitcoin cash
4	monero	litecoin
5	lisk	cardano
6	bitcoin gold	stellar
7	tether	eos
8	steem	iota
9	populous	nem
10	siacoin	ethereum classic
⋮	⋮	⋮

The first 10 predictor and response variables in order of capitalization, selected using the heuristic criterion given above. The total number of selected variables is shown in parentheses.

### Modularity and centrality

To confirm that this variable selection makes sense, we have found the structure of the associated clusters through the modularity approach. This technique is based on the principle that networks that tend to form clusters differ from random networks [48]. It measures the deviation from a random network for a specific partition of the nodes into clusters. Then, we find the optimal partition maximizing the modularity version for directed networks [49] defined by

$$\begin{array}{c} Q=\frac{1}{N_{E}}\sum_{i,j}[A_{ij}-\frac{k_{i}^{out}k_{j}^{in}}{N_{E}}\delta \left(c_{i},c_{j}\right)], \end{array}$$ (13)

where *A*_*ij*_ is the binary entry of the adjacency matrix, which represents the existence or not of an edge between nodes *i* and *j* by one or zero, respectively; *c*_*i*_ is the label of the cluster to which node *i* is assigned to, and *N*_*E*_ is the total number of edges in the network. Then, the implicit idea behind is to maximize *Q* over possible divisions of the network into clusters or *communities*.

**Fig 5:**
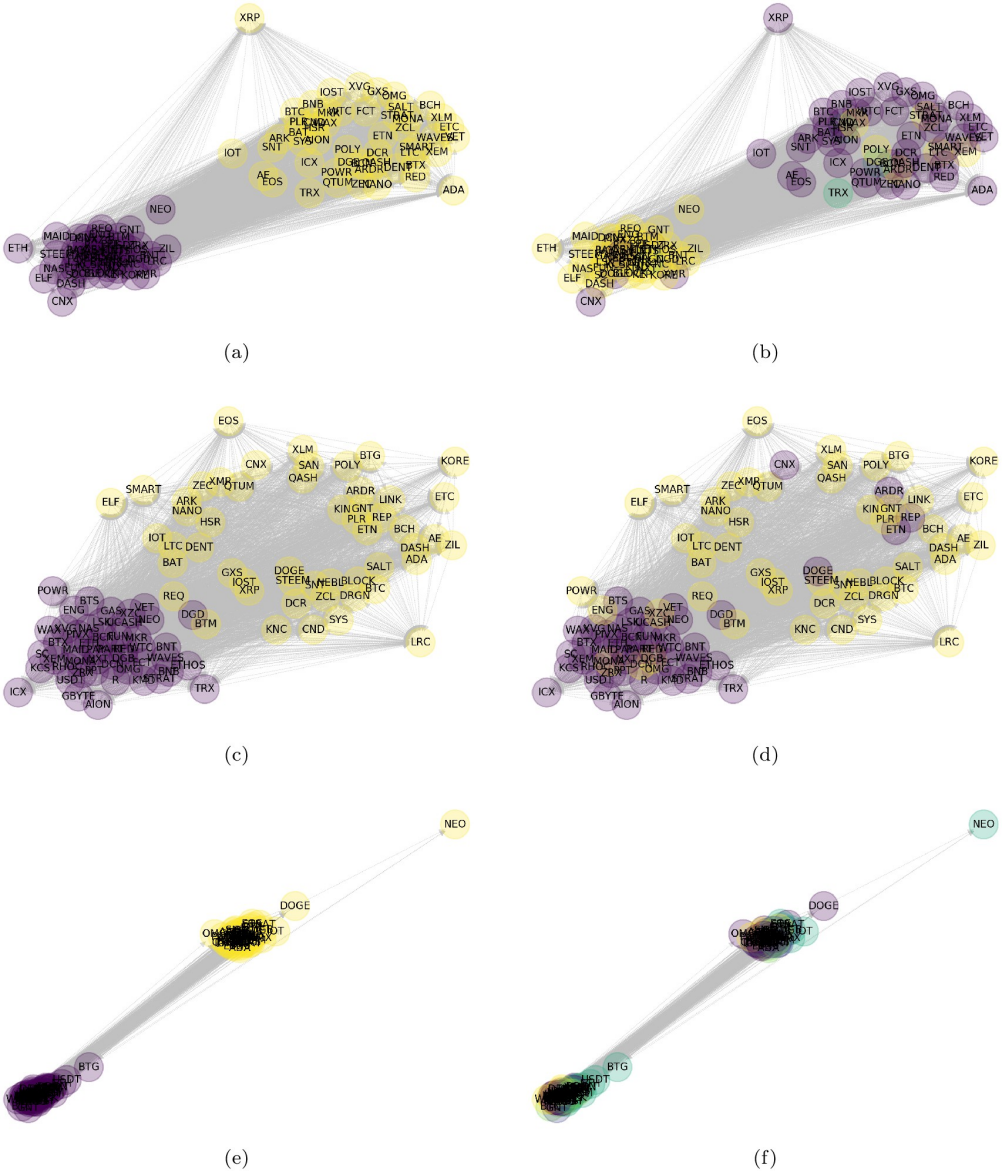
Predictor-reponse clusters vs. modularity clusters. Comparision of the cluster formed by the heuristic variable selection rule of Eq 12 (left figures) and the cluster formed by the modularity approach of Eq 13 (right figures). From top to bottom the cases Δ*t* = 1, *m* = 3((a) and (b)), Δ*t* = 1, *m* = 2 ((c) and (d)), Δ*t* = 0, *m* = 4 ((e) and (f)). The node names represent the symbol of the associated cryptocurrency as listed on the annexed table in the appendix (see S1 Table).

In the top graphs of Fig 5 it is shown the generated clusters via our heuristic approach (Fig 5a) and the optimal generated clusters under the modularity approach (Fig 5b), both results for the case *Deltat* = 1, *m* = 3. In spite of the fact that under the modularity approach the NDI network form three clusters, they resemble our predictor and response sets. It is worth to mention that the visualization is made using the Force-directed algorithm [50]. Here, the position of each node is fixed by considering the weights of the edges as an attractive spring force and is seek equilibrium of forces in each node by adding an electrostatic repulsive force as a free parameter. In addition, we have separated the clusters from its equilibrium position for visualization purposes by a radial distance of $\sqrt{1/2}$ to the top-right direction if the node belongs to the response set, and to the same distance but to the bottom left direction if the node belongs to the predictor set under. In this manner, the node position does not change in both results since the algorithm only considers the associated weights to fix the position. On the contrary, the color changes according to the cluster at which each node corresponds.

A surprising result is that *ripple* is consistently assigned by modularity to a cluster with a similar pattern as the response set. This behavior is interesting because ripple looks quite different from all other cryptocurrencies in the force-directed visualization. A possible explanation is due to the particular characteristics of *ripple* as a digital payment protocol and its increasing adoption by banks for its faster international transactions over the traditional swift payment method.

In the middle graphs of Fig 5 it is shown the equivalent analysis of above for the case Δ*t* = 1, *m* = 2, where we can see that the variable selection nodes via our heuristic rule are not completely separated in the sense of forces (Fig 5c), but in this case the modularity approach found two clusters (Fig 5d). To contrast this behavior, in the bottom graphs of Fig 5 we have shown one of the worst cases under our heuristic rule, corresponding to Δ*t* = 0, *m* = 4, where #{$T_{ab}^{S}>0$} has the lower value in Table 1. Here we can see that the external force of $\sqrt{2}$ applied to separate the sets or response and predictor nodes is stronger than the internal force between the nodes, as a consequence, a pattern is more difficult to find. This can be confirmed by the modularity approach, which has found five clusters in this case.

In Table 3 we show the percentage of identification of each cluster found by modularity in relation to the predictor and response variable sets categorized under our selection rule. It can be seen case Δ*t* = 1, *m* = 2 has the best identification, where the elements of cluster one given by modularity coincide with the elements of the predictor set in an 87.5%, while cluster two coincide with the response set in a 90.38%. Similarly, our preferred case of Δ*t* = 1, *m* = 3 have a coincide of 83.67% and 82.35%, respectively. Nevertheless the case Δ*t* = 1, *m* = 2 have higher coincide percentage, we still prefer the case Δ*t* = 1, *m* = 3 over it as the best partition because the larger separation of the sets under the force-directed algorithm (Fig 5a and 5b).

**Table 3:** Cluster identification.

**Δt = 1, m = 3**	**Predictor (49)**	**Response (51)**
Cluster 1	83.67%	11.76%
Cluster 2	12.25%	82.35%
Cluster 3	4.08%	5.88%
**Δt** = **1**, **m** = **2**	**Predictor (48)**	**Response (52)**
Cluster 1	87.5%	9.61%
Cluster 2	12.5%	90.38%
**Δt** = **0**, **m** = **4**	**Predictor (56)**	**Response (44)**
Cluster 1	14.29%	22.73%
Cluster 2	28.57%	31.82%
Cluster 3	25%	15.91%
Cluster 4	21.43%	20.46%
Cluster 5	10.71%	9.09%

Percentage of coincidence between the predictor-response sets and the clusters found by the modularity approach for different values of Δ*t* and *m*.

To explore further the structure of the directed network associated with NDI of the transfer entropy, we have computed the in-degree and out-degree centralities of the whole network. The in-degree centrality for a node *V* is defined as the fraction of nodes in which incoming edges are connected to. The out-degree centrality for a node *V* is the fraction of nodes whose outgoing edges are connected to. Before showing the results, it is important to mention that cryptocurrencies are divided into two main categories: coins and tokens. Basically, coins have the same characteristics as money. i.e., they are fungible, divisible, acceptable, portable, durable and have limited supply, while tokens are issued by the project, which can be used as a method of payment inside the project’s ecosystem, in this way they are more like digital assets. Tokens, unlike coins, are created on top of existing blockchains. By far the most common platform to create tokens is the Ethereum platform. Other token platforms include Stellar, NEO, Omni, and EOS.

In Table 4 it is shown the top ten in-degree and out-degree centrality ranking of cryptocurrencies, from the highest to the lowest. The first and fourth column represents the order number in terms of capitalization, the second and fifth the name of the cryptocurrency, the third and sixth the type, i.e., coin or token, and finally, the seventh column specify from which platform was created the token. The results show that the highest in-degree centrality values are dominated by coins, while mainly tokens have the highest out-degree values. This result tells us that coins are central in the sense of receiving information, while tokens are central in the sense of sending information. Hereof, the direction of information transfer helps to discriminate between tokens and coins.

**Table 4:** In-degree and out-degree centrality.

in-degree			out-degree			
number	name	type	number	name	type	platform
2	ripple	coin	42	komodo	coin	
8	eos	coin	62	kyber network	token	ethereum
14	ethereum classic	coin	81	funfair	token	ethereum
5	cardano	coin	52	digixdao	token	ethereum
3	bitcoin cash	coin	72	ethos	token	ethereum
20	icon	coin	94	bancor	token	ethereum
9	iota	coin	13	lisk	token	ethereum
23	raiblocks	coin	53	gas	token	NEO
91	reddcoin	coin	38	0x	token	ethereum
22	zcash	coin	18	bitcoin gold	coin	

Top ten in-degree and out-degree centrality ranking of cryptocurrencies.

In some networks, it is appropriate also to accord a vertex high centrality if it points to others with high centrality. There are two types of important nodes in this context: authorities and hubs. Authorities are nodes that contain useful information on a topic of interest; hubs are nodes that tell us where the best authorities are to be found [51]. Specifically, authority centrality is defined as the sum of the hub centralities *y*_*j*_ which point to the node *x*_*i*_

$$\begin{array}{c} x_{i}=\alpha \sum_{j}^{p}A_{ij}y_{j}, \end{array}$$ (14)

for *i* = 1, …, *p*, where *α* is constant. Likewise, Hub Centrality is the sum of the authorities *x*_*j*_ which point to the node *y*_*i*_

$$\begin{array}{c} y_{i}=\beta \sum_{j}^{p}A_{ij}x_{j}, \end{array}$$ (15)

for *i* = 1, …, *p*, with constant *β*. In matrix notation

$$\begin{array}{c} \text{x}=\alpha \text{Ay},\;\text{y}=\beta {\text{A}}^{\prime }\text{x} \end{array}$$ (16)

These expressions were proposed and developed by Kleinberg [52] into a centrality algorithm called hyperlink-induced topic search or HITS.

In Table 5 it is shown the top ten hub and authority centrality ranking of cryptocurrencies, from the highest to the lowest. The first and fifth column represents the order number in terms of capitalization, the second and sixth the name of the cryptocurrency, the third and seventh the type, i.e. coin or token, and finally, the fourth and eighth column specify from which platform was created the token. The results show that there is not a clear pattern in the hub centrality values, but authorities are dominated by coins. This can be interpreted as coins are central authorities of the transfer entropy network in the sense that they are pointed in by a lot of good hub cryptocurrencies. This means that coins are authorities in the ecosystem of cryptocurrencies.

**Table 5:** Hubs and authorities.

Hubs				Authorities			
number	name	type	platform	number	name	type	platform
98	particl	coin		91	reddcoin	coin	
82	kin	coin		63	monacoin	coin	
95	tenx	token	ethereum	80	dent	token	ethereum
90	chainlink	token	ethereum	2	ripple	coin	
87	neblio	coin		73	pillar	token	ethereum
67	dentacoin	token	ethereum	23	raiblocks	coin	
50	revain	token	ethereum	77	factom	coin	
42	komodo	coin		48	zclassic	coin	
97	santiment	token	ethereum	22	zcash	coin	
54	byteball	coin		14	ethereum_classic	coin	

Top ten Hub and Authority centrality ranking of cryptocurrencies.

## Regression framework

Now that the set of predictor-response variables has been determined and analyzed, we would like to present a general regression model as the framework to forecast the response variables. Thus, this section describes the model and the related problem of rank determination that highlights the need to study some results from random matrices.

Consider the reduced-rank regression (RRR) model given by [53]

$$\begin{array}{c} \overset{s\times 1}{\text{Y}}=\overset{s\times 1}{\mu }+\overset{s\times r,}{\text{C}}\overset{r\times 1}{\text{X}}+\overset{s\times 1}{\varepsilon } \end{array}$$ (17)

where *μ* and **C** are unknown regression parameters, and the unobservable error variate *ε* of the model has mean *E*(*ε*) = 0 and covariance matrix *cov*(*ε*) = *E*{*εε*′} = Σ_*εε*_, and is distributed independently of **X**. The difference from the classical multivariate regression model is that the rank of the regression coefficient matrix **C** is deficient

$$\begin{array}{c} \text{rank}(\text{C})=\eta \leq \text{min}(r,s). \end{array}$$ (18)

The rank condition implies that there may be a number of linear constraints on the set of regression coefficients in the model. Given a sample **X**,**Y** of observations, the goal is to estimate optimally the parameters *μ* and **C**. Hence, the idea is to minimize the objective function

$$\begin{array}{c} W\left(t\right)=E\{{\left(\text{Y}-\mu -\text{CX}\right)}^{\prime }\Gamma \left(\text{Y}-\mu -\text{CX}\right)\}, \end{array}$$ (19)

where **Γ** is a positive definite symmetric matrix of weights, and the expectation is taken over the joint distribution of **X**,**Y**.

RRR can be regarded as a unifying treatment of several classical multivariate procedures that were developed separately from each other. If we set **X** (and *r* = *s*) by making the output variables identical to the input variables and, in addition, set **Γ** = **I**, then we obtain Harold Hotelling’s PCA and exploratory factor analysis. If we set $\Gamma =\Sigma_{\text{YY}}^{-1}$, then we have Hotelling’s canonical variance and correlation analysis. A nonlinear generalization of RRR provides a flexible model for artificial neural networks [54]. Nevertheless, one of the primary and most difficult parts of model determination is to assess the unknown value of parameter *η*, called the effective dimensionality of the multivariate regression. The reduction in *W*_*min*_(*η*) obtained by increasing the rank from *η* = *η*_0_ to *η* = *η*_1_, where *η*_0_ < *η*_1_, is given by

$$\begin{array}{c} W_{\text{min}}\left(\eta_{0}\right)-W_{\text{min}}\left(\eta_{1}\right)=\sum_{j=\eta_{0}+1}^{\eta_{1}}\lambda_{j}. \end{array}$$ (20)

This relation depends upon **Γ** only through the eigenvalues {λ_*j*_} of

$$\begin{array}{c} \text{N}=\Gamma \Sigma_{\text{YX}}\Sigma_{\text{XX}}^{-1}\Sigma_{\text{XY}}\Gamma \end{array}$$ (21)

However, the value of *η* and, hence, the number and nature of those constraints may not be known prior to statistical analysis.

We are interested in estimate the parameter *η* for the particular case of $\Gamma =\Sigma_{\text{YY}}^{-1}$, i.e., the canonical correlation analysis, and explore the properties and behavior of the number of factors through time. Our focus is on the high-dimensional settings under the random matrices approach. Hence, in the next section, we introduce the theory step-by-step and describe the results for the predictor-response sets of cryptocurrencies. The core of the technique is the deep connection of the estimation of *η* via the Tracy-Widom distribution as we will see in what follows.

## Number of factors

The random matrix theory (RMT) is an important framework for analyzing limit distributions of eigenvalues. Historically, RMT was developed to solve complex problems in nuclear physics and more recently in quantum chaos [55]. During the preceding decades, seminal applications of RMT arose in the context of mesoscopic physics, biological microarrays, wireless communication and econophysics [56–60]. A common ingredient of the cited studies is the following result, restated here using the terminology of high-dimensional statistics.

Let *X* be a *p* × *n* matrix, where the elements *X*_*i*,*j*_ are i.i.d. random variables with distribution *N*(0, 1). Then, as *p*, *n* → ∞, such that $\frac{n}{p}\to c\in \left(0,\infty \right)$, the spectral density of the Wishart matrix $W=n^{-1}XX^{{}^{\prime }}$ converges (a.s.) to the Marcenko-Pastur law [25]

$$\begin{array}{c} \rho \left(\lambda \right)=\frac{\sqrt{\left(\lambda_{max}-\lambda \right)\left(\lambda -\lambda_{min}\right)}}{2\pi c\lambda }, \end{array}$$ (22)

where

$$\begin{array}{c} \lambda_{min}^{max}={\left(1\pm \sqrt{c}\right)}^{2}. \end{array}$$ (23)

In the econophysics community, the Marchenko-Pastur distribution is known as a universal result of Wishart matrices. If there is no correlation between financial variables, then the eigenvalues of their correlation matrix should be bounded by this RMT prediction [59, 60].

In statistics, it is of primary importance to consider null hypothesis tests. The Wishart matrices that appear in the preceding result can be denoted by *W*_*p*_(*n*, I), where I is the covariance matrix of the population distribution of *n*^−1^ *XX*′. In our case, it is of interest to test the hypothesis of identity covariance matrix, *H*_0_: Σ = I, against an alternative case *H*_*A*_: Σ ≠ I, where Σ has some more general structure. Using this approach, it is possible to compute a confidence interval for accepting or rejecting the universal result of Wishart matrices of empirical datasets for the general range of dimensions *p* and *n*. The approach to quantifying a confidence level is based on the approximation to the null hypothesis’ distribution of the largest sample eigenvalue ${\hat{\lambda }}_{1}$

$$\begin{array}{c} P\{{\hat{\lambda }}_{1}>M:W∼W_{p}\left(n,I\right)\}, \end{array}$$ (24)

which is the probability of finding $\hat{\lambda }$ larger than an upper bound M, given that *W* = *n*^−1^ *XX*′ follows the Wishart distribution *W*_*p*_(*n*, I). The following result of the random matrix theory leads to the needed approximate distribution [10].

Assume *A* ∼ *W*_*p*_(*n*, I), *p*/*n* → *γ* ∈ (0, ∞), and denote by ${\hat{\lambda }}_{1}$ the largest eigenvalue in the eigenvalue equation $Au=\hat{\lambda }u$. Then, the distribution of the largest eigenvalue approaches that of the Tracy–Widom *F*_*β*_ law

$$\begin{array}{c} P\left\{n{\hat{\lambda }}_{1}\leq \mu_{np}+\sigma_{np}s|H_{0}\right\}\to F_{\beta }\left(s\right) \end{array}$$ (25)

where $\mu_{np}={\left(\sqrt{n}+\sqrt{p}\right)}^{2}$, $\sigma_{np}=\mu_{np}{\left(\frac{1}{\sqrt{n}}+\frac{1}{\sqrt{p}}\right)}^{1/3}$. There are elegant formulas for computing the Tracy-Widom distribution functions:

$$\begin{array}{c} \begin{array}{cc} F_{1}\left(s\right) & =\sqrt{F_{2}\left(s\right)\exp(-\int_{s}^{\infty }q\left(x\right)dx)}\\ F_{2}\left(s\right) & =\exp(-\int_{s}^{\infty }{\left(x-s\right)}^{2}q\left(x\right)dx), \end{array} \end{array}$$ (26)

in terms of the solution *q* of a nonlinear second-order differential equation *q*″ = *sq* + 2*q*^3^, *q*(*s*)∼*A*_*i*_(*s*) as *s* → ∞, also known as the classical Painlevé type II equation. Functions in the family *F*_*β*_ are obtained numerically as a function of *q*. Even though some effort is required to solve for *F*_*β*_, from the point of view of applied data analysis, they are special functions, as is the normal curve [61].

Let us provide an example of the relevance of the Tracy-Widom test. Suppose that in a sample of *n* = 10 observations from a *p* = 10-dimensional Gaussian distribution *N*_10_(0, Σ), the largest sample eigenvalue λ_1_ = 4.25 has been obtained. Given these dimensions, the support of the Marchenko-Pastur distribution is bounded to the interval [0, 4] (see Eq (23)) Then, the question in statistical terms is whether an observed largest eigenvalue of 4.25 is consistent with *H*_0_: Σ = I if *n* = *p* = 10. The second-order Tracy–Widom approximation [62] yields 6% chance of observing a value more extreme than 4.25 even if no structure is present, i.e., Σ = I. Considering the traditional 5% benchmark, this is not strong enough evidence to reject the null hypothesis *H*_0_ [63].

The Tracy-Widom test becomes relevant to the determination of the number of components that must be retained in PCA, especially in the context of high-dimensional data, i.e., if O(n/p)=1. Beyond PCA, there are several classical problems in multivariate statistics that can take advantage of this type of test. These problems can be generalized under the greatest root distribution. It describes the null hypothesis of apparently different problems, including multiple-response linear regression, multivariate analysis of variance, canonical correlations, equality of covariance matrices, etc. [64]. The next definition from [65] states the greatest root distribution formally.

Let **A** ∼ *W*_*p*_(*l*, **I**) be independent of **B** ∼ *W*_*p*_(*n*, **I**), where *l* ≥ *p*. Then, the largest eigenvalue *θ* of (**A** + **B**)^−1^**B** is called the greatest root statistic, and its distribution is denoted by *θ*(*p*, *l*, *n*). It has the property

$$\begin{array}{c} \theta \left(p,l,n\right)\overset{d}{=}\theta \left(n,l+n-p,p\right), \end{array}$$ (27)

that is useful, in particular, if *n* < *p*.

There is an interesting connection between the greatest root statistic and Tracy-Widom distributions. In the study of Johnstone [13], it is shown that with appropriate centering and scaling, the distribution of the logit transform *W* of *θ* approximates a Tracy-Widom distribution:

$$\begin{array}{c} \frac{W(p,l,n)-\mu (p,l,n)}{\sigma (p,l,n)}\overset{d}{\to }F_{1}, \end{array}$$ (28)

where

$$\begin{array}{c} W\left(p,l,n\right)=\text{logit}\theta \left(p,l,n\right)=\log(\frac{\theta (p,l,n)}{1-\theta (p,l,n)}) \end{array}$$ (29)

is the logit transform of *θ*, and the centering and scaling parameters are defined by

$$\begin{array}{c} \mu \left(p,l,n\right)=2\log\tan(\frac{\phi +\gamma }{2}),\sigma^{3}\left(p,l,n\right)=\frac{16}{{\left(l+n-1\right)}^{2}}\frac{1}{\sin^{2}\left(\phi +\gamma \right)\sin\phi \sin\gamma }, \end{array}$$ (30)

where the angle parameters *γ*, *ϕ* are defined as

$$\begin{array}{c} \sin^{2}(\frac{\gamma }{2})=\frac{min(p,n)-1/2}{l+n-1},\sin^{2}(\frac{\phi }{2})=\frac{max(p,n)-1/2}{l+n-1}. \end{array}$$ (31)

At this point, we are interested in describing a procedure for determining parameter *η* in the RRR model by using the greatest root statistic, which will reconcile both frameworks. This is traditionally accomplished by the canonical correlation analysis (CCA). The respective approach involves partitioning a collection of variables into two sets. Consider a set **X** with *q* variables and a set **X** with *p* variables. The objective is to find maximally correlated combinations *η* = **a**′**x** and *ϕ* = **b**′**y**. Even though CCA has maximal properties similar to those of PCA, the objective of canonical correlation concerns the relationship between two groups of variables instead of interrelationships within a set of variables.

Suppose that (**X**,**Y**) is a data matrix of *n* observations on *q* + *p* variables such that each sample is independent of the others and has the populations distribution *N*_*p*+*q*_(*μ*,**Σ**). Assume the sample covariance matrix **S** partitioning

$$\begin{array}{c} \text{S}=(\begin{array}{cc} {\text{S}}_{\text{XX}} & {\text{S}}_{\text{XY}}\\ {\text{S}}_{\text{YX}} & {\text{S}}_{\text{YY}} \end{array}). \end{array}$$ (32)

The sample squared canonical correlations ($r_{i}^{2}$) for *i* = 1, …, *k* = min(*p*, *q*) are obtained as the eigenvalues of ${\text{M}}_{\text{S}}={\text{S}}_{\text{YY}}^{-1}{\text{S}}_{\text{YX}}{\text{S}}_{\text{XX}}^{-1}{\text{S}}_{\text{XY}}$, whereas the population counterparts are given by the eigenvalues of ${\text{M}}_{\Sigma }=\Sigma_{\text{YY}}^{-1}\Sigma_{\text{YX}}\Sigma_{\text{XX}}^{-1}\Sigma_{\text{XY}}$ [65]. Note that the nonzero eigenvalues of **M**_**Σ**_ are the same as the nonzero eigenvalues of **N** in Eq (21) for $\Gamma =\Sigma_{\text{YY}}^{-1}$, which is precisely the CCA case in the general RRR model.

We are now interested in describing the procedure of testing the null hypothesis of independence of the two sets of variables *H*_0_: **Σ**_**12**_ = 0 by using the Tracy-Widom test. First, let us point out the next result concerning joint independence of partitioned Wishart matrices.

Let **M** ∼ *W*_*p*_(*n*, **Σ**), and partition matrix **M** into submatrices **M**_11_ of dimensions *a* × *a* and **M**_22_ of dimensions *b* × *b*, where *a* + *b* = *p* and *n* > *a*. Define the product of matrices ${\text{M}}_{3}={\text{M}}_{22}-{\text{M}}_{21}{\text{M}}_{11}^{-1}{\text{M}}_{12}$. Then [65],

1. **M**_3_ has the distribution *W*_*b*_(*n* − *a*, **Σ**_**3**_) and is independent of (**M**_11_, **M**_22_),
2. if **Σ**_12_ = 0, then ${\text{M}}_{22}-{\text{M}}_{3}={\text{M}}_{21}{\text{M}}_{11}^{-1}{\text{M}}_{12}$ has the distribution *W*_*b*_(*a*, **Σ**_22_), and ${\text{M}}_{21}{\text{M}}_{11}^{-1}{\text{M}}_{12}$, **M**_11_, and **M**_3_ are jointly independent.

On the other hand, the hypothesis technique of the Union Intersection Test (UIT) uses the statistic based on the largest eigenvalue $r_{1}^{2}$ of **M**_**S**_.

However, **M**_**S**_ can be written as [**M**_3_ + (**M**_22_ − **M**_**3**_)]^−1^(**M**_22_ − **M**_3_), where **M**_22_ = *n***S**_*YY*_, ${\text{M}}_{3}=n\left({\text{S}}_{\text{YY}}-{\text{S}}_{\text{YX}}{\text{S}}_{\text{XX}}^{-1}{\text{S}}_{\text{XY}}\right)$, and **M**_22_ − **M**_3_ satisfies the independence condition of the greatest root statistic. Therefore, under *H*_0_: **Σ**_**12**_ = 0, $r_{1}^{2}$ has the distribution *θ*(*p*, *n* − *q* − 1, *q*), and the Tracy-Widom approximation can be applied.

The previous derivation shows a procedure for statistically determining the rank *η* of an RRR model through the RMT framework. Specifically, it has established the connection of the Tracy-Widom distribution to testing the null hypothesis *H*_0_: **Σ**_**12**_ = 0 in the particular case of CCA for general RRR models. In what follows, the applied methodology for finding the number of significant components or factors in the CCA using our datasets of predictor and response cryptocurrency variables is described.

### Analysis of predictor-response cryptocurrency factors

The first step in using these techniques with real-world data is based on numerically solving the system of equations in Eq (26), taking into account the Painlevé equations with the boundary condition that as *t* → ∞, *q*(*t*) asymptotically converges the Airy function *Ai*(*t*). We solve these nonlinear differential equations with an relative tolerance error of 1 × 10^−12^, following the approach described in [61]. Table 6 shows a subsample of the obtained values. The first and second columns display values of x,y on the plane of the Tracy-Widom distribution, respectively. The third column shows the cumulative density value (cdv) corresponding to the respective values of x,y; the result of subtracting that value from 1 determines the level of significance in the statistical test of Tracy-Widom.

**Table 6:** Tracy-Widom values.

x	y	cdv
⋮	⋮	⋮
1.995	0.017669	0.989510
2.000	0.017535	0.989598
2.005	0.017402	0.989685
2.010	0.017270	0.989771
2.015	0.017139	0.989857
2.020	0.017009	0.989942
2.025	0.016880	0.990026
2.030	0.016751	0.990110
2.035	0.016623	0.990193
2.040	0.016497	0.990276
⋮	⋮	⋮

Subsample of values *x*, *y* on a plane and the corresponding cdv of the Tracy-Widom distribution.

Next, we apply CCA to the set of cryptocurrency variables. In this analysis, the predictor and response variables previously obtained in the section on variable selection are regarded as sets **X**,**Y**, respectively, where the response variables **Y** are led by a time step of Δ*t* = 1 hour, as they were in the variable selection analysis. However, when applying the standard CCA, we observed numerical instabilities because some underlying variables were nearly collinear. To avoid this issue, the regularized version of the CCA is applied [66]. In this approach, covariance matrices **S**_**XX**_ and **S**_**YY**_ are replaced by

$$\begin{array}{c} \begin{array}{cc} & {\text{S}}_{\text{XX}}+\lambda I_{x}\\ & {\text{S}}_{\text{YY}}+\lambda I_{y}, \end{array} \end{array}$$ (33)

where I_x_, I_y_ are identity matrices of the same dimensions as the corresponding covariance matrices **S**_**XX**_,**S**_**YY**_, and λ is a regularization parameter. Applying the regularized CCA ensures that the relevant covariance matrices have numerically stable inverse matrices. This transformation only affects the eigenvalues, which suffer a translation by the same amount λ, but the eigenvectors remain unchanged. Accordingly, we compute the eigenvalues of matrix **M**_**s**_, considering a regularization parameter λ = 0.01. Then, under the greatest root distribution *θ*(*p*, *n* − *q* − 1, *q*) with parameters *p* = 49 (the number of predictor variables), *q* = 51 (the number of response variables), and *n* = 4531 (the number of observations after the time horizon reduction by considering lag-lead returns) using Eqs (28)–(31) results in obtaining 36 factors explaining 93.59% of variance at the significance level of *α* = 0.01.

**Fig 6:**
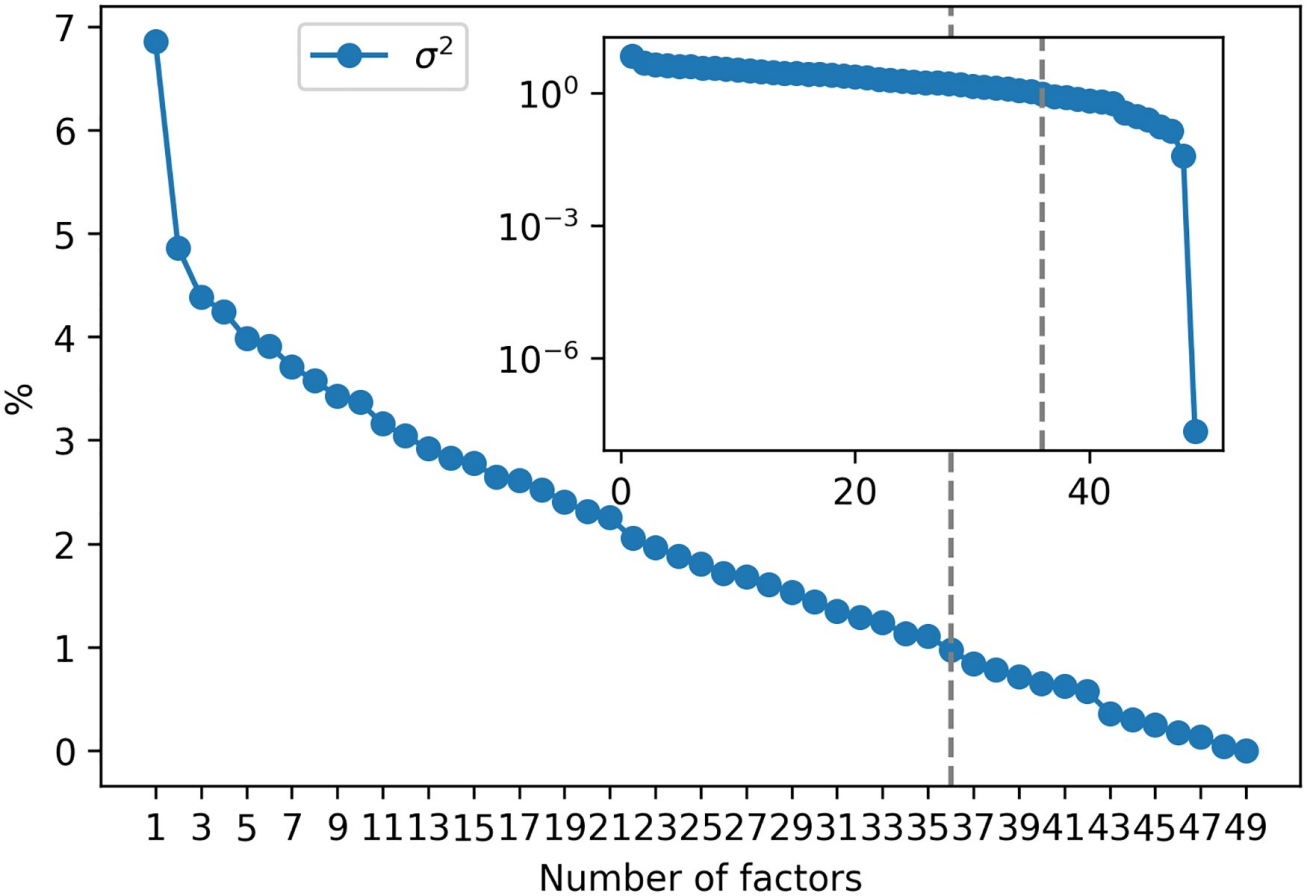
Explained variance in CCA. Percentage contribution to variance as a function of the component. The inset graph show the same but on a semi-log scale.

Fig 6 shows the percentage of explained variance as a function of the number of factors. Although, in CCA, the increments of the predictor and response components are considered symmetrically, the fixed lag time of Δ*t* = 1 provides the predictive element. The dashed vertical gray line represents the cutoff point where the number of significant components is determined. The inset graph shows the same but on a semi-log scale. The plot does not show an abrupt change in the curve. Hence, if we use the elbow criterion, it would not be possible to determine the appropriate number of components to be considered in the model. Moreover, compared with the resampling approach, the computational time of the Tracy-Widom test is negligible since we only need to compute the table of the significance level once.

In addition, Table 7 shows the number of factors and percentage of explained variance for λ ∈ {0.1, 0.01, 0.001, 1 × 10^−4^, 1 × 10^−5^} and *α* ∈ {0.1, 0.01, 0.001}. It is observed the number of factors oscillates between 34 and 37, while the corresponding explained variance is between 91.42% and 94.56%. Additionally, the number of factors seems to reach a stable value of 36 as λ, *α* become smaller.

**Table 7:** Number of factors as a function of λ and *α*.

λ	*α*	Number of factors	Explained variance (%)
0.1	0.1	35	92.54
0.1	0.01	35	92.54
0.1	0.001	34	91.42
0.01	0.1	36	93.59
0.01	0.01	36	93.59
0.01	0.001	35	92.48
0.001	0.1	37	94.56
0.001	0.01	36	93.59
0.001	0.001	36	93.59
1 × 10^−4^	0.1	37	94.56
1 × 10^−4^	0.01	36	93.59
1 × 10^−4^	0.001	36	93.59
1 × 10^−5^	0.1	37	94.56
1 × 10^−5^	0.01	36	93.59
1 × 10^−5^	0.001	36	93.59

Number of factors and the respective explained variance (as a percentage) for various values of the regularization parameter λ and significance level *α*.

Furthermore, the dynamic behavior of the number of factors can be analyzed. To this end, we partition the whole sample period and choose a set of subsample data matrices for four weeks (*n* = 672 hours) and a moving window of one week (168 hours). As a result, we obtain *m* = 23 subsample data matrices to which CCA is applied for the set of predictor-response variables obtained in the section on variable selection while considering the entire period of time.

**Fig 7:**
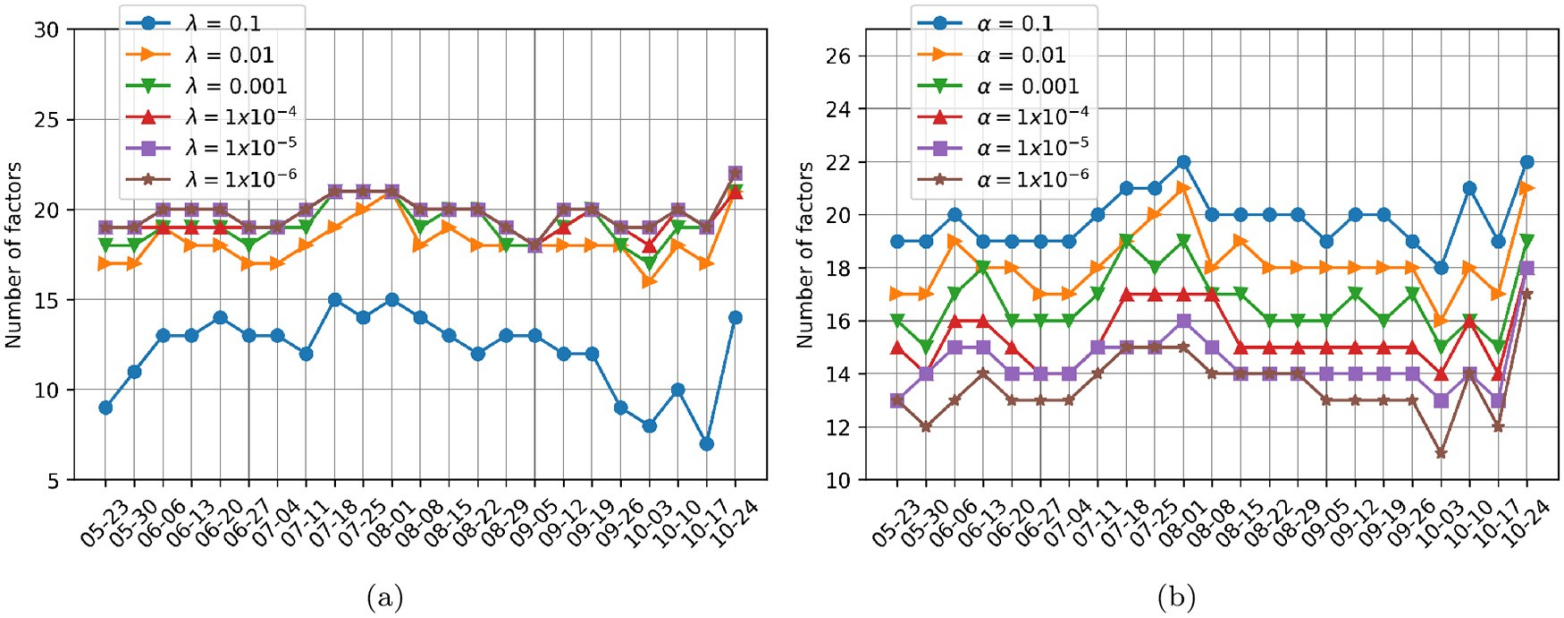
Number of factors as a function of time. Estimated factors for subsample data matrices over four weeks with a moving window of one week. (a) The level of significance is set at *α* = 0.01, and the regularization parameter λ is varied. (b) The regularization parameter is set at λ = 0.01, and the significance level *α* is varied.

Fig 7a shows the estimated number of factors as a function of the moving window for a fixed level of significance *α* = 0.01 and various values of the regularization parameter λ ∈ {0.1, 0.01, 0.001, 1 × 10^−4^, 1 × 10^−5^, 1 × 10^−6^}, whereas Fig 7b presents the same analysis, but for a fixed value of λ = 0.01 and various levels of significance *α* ∈ {0.1, 0.01, 0.001, 1 × 10^−4^, 1 × 10^−5^, 1 × 10^−6^}. In all cases, the estimation of the number of factors was performed using the same proposed procedure as in the analysis of the entire period (Eqs (28)–(31)). We observe in Fig 7a that the number of factors increases as the value of λ decreases, reaching a stable behavior at λ = 1 × 10^−5^, where the number of factors does not change for smaller values of λ and is thus similar to that observed at λ = 1 × 10^−6^. In contrast, in Fig 7b, the number of factors decreases as the value of *α* decreases, but the temporal behavior seems to have the same tendency as in Fig 7a. The fact that the number of factors decreases with the contraction of the time horizon is a natural behavior explained by Harding in [11]. Nonetheless, all cases exhibit a high value on November 24, 2018 independent of the magnitude.

To investigate a possible relation between the dynamics of the number of factors and the return of the exchange value of cryptocurrencies, two indices were constructed from the predictor and response variables. Let *I*_*x*_(*l*) be the composite index constructed by averaging over all the predictor variables during subsample periods *l* = 1, …, *m*. Similarly, we denote by *I*_*y*_(*l*) the composite index constructed in the same manner, but considering the response variables. Table 8 shows the correlation values between these composite indices in relation with the variation of the number of factors as a function of time. We observe a moderate anticorrelation in all cases, ranging from −0.29 to −0.52. This result suggests that the variation in the number of factors over time is influenced by the behavior of the composite returns of the predictor and response cryptocurrencies. This result can be used in future work to modeling the collective behavior of the cryptocurrency market under a predictive model using as a predictor the time series created by the variation of the number of factors through time.

**Table 8:** Correlations between *I*_*x*_, *I*_*y*_ and the number of factors.

λ	*α*	Corr(*I*_*x*_, #factors)	Corr(*I*_*y*_, #factors)
0.1	0.01	-0.29	-0.27
0.01	0.01	-0.45	-0.44
0.001	0.01	-0.40	-0.41
1 × 10^−4^	0.01	-0.43	-0.45
1 × 10^−5^	0.01	-0.43	-0.44
1 × 10^−6^	0.01	-0.43	-0.44
0.01	0.1	-0.42	-0.41
0.01	0.01	-0.45	-0.44
0.01	0.001	-0.46	-0.45
0.01	1 × 10^−4^	-0.52	-0.51
0.01	1 × 10^−5^	-0.49	-0.46
0.01	1 × 10^−6^	-0.47	-0.45

Pearson correlations between the composite indices *I*_*x*_, *I*_*y*_ and the number of factors (denoted by #factors). The same values of λ and *α* as in Fig 6 are considered.

**Fig 8:**
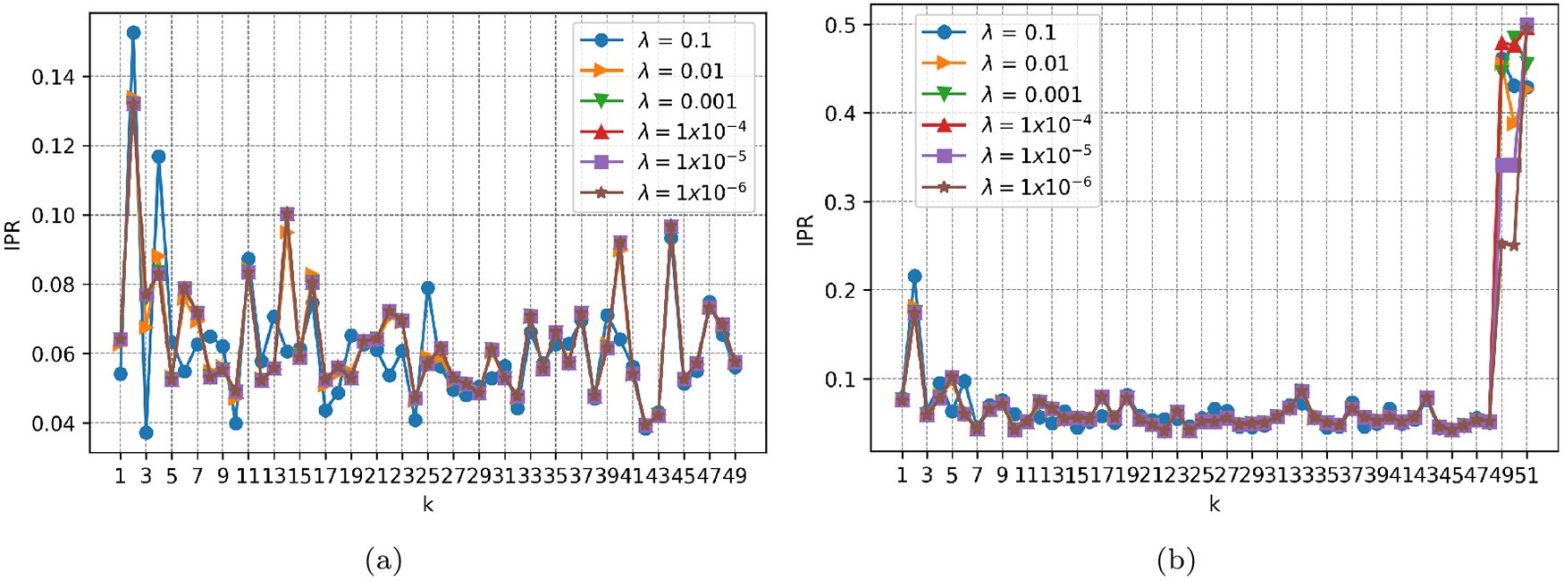
IPR. Inverse participation ratio for the entire sample period of time for various values of the regularization parameter λ. (a) Predictor factors. (b) Response factors.

To identify the compositions of the predictor-response factors, i.e., if the weights contribute equally or if there are peaks where some cryptocurrencies have a dominant contribution, we compute the inverse participation ratio (IPR). This measure originates from physics, where it is used as a measure of localization [67]; however, in the context of econophysics, it allows us to determine the number of components that participate significantly in each eigenvector associated with the predictor-response factors. The IPR of eigenvector *V*_*k*_ is given by

$$\begin{array}{c} IPR\left(k\right)=\sum_{j=1}^{r}{\left|V_{k}\left(j\right)\right|}^{4}, \end{array}$$ (34)

where *k* = 1, …, *r*, and *r* = *p* or *q*, depending on whether eigenvectors *V*_*k*_ are associated with the predictor or response factors, respectively. This quantity is bounded between 1/*r* and 1. If eigenvector *V*_*k*_ is localized only in one component, or equivalently, if only one coin has a dominant weight contribution, then *IPR*(*k*) = 1. In contrast, if *V*_*k*_ is not localized or is uniformly distributed over *r* weights, then *IPR*_*k*_ = 1/*r*. In Fig 8, we plot IPR for (a) the predictor factors and (b) the response factors of the entire sample period, varying the regularization parameter λ ∈ {0.1, 0.01, 0.001, 1 × 10^−4^, 1 × 10^−5^, 1 × ^1^ 0^−6^} and setting the level of significance to *α* = 0.01. We do not change *α* in this analysis because doing so does not alter the results. We observe first that the behavior of IPR does not change significantly for either predictors or responses as λ is varied. A relevant point is that by considering IPR we can distinguish between the *localized factors*, i.e., those that have dominant weights related to specific cryptocurrencies, and the *nonlocalized factors* that have uniform coin contributions.

**Fig 9:**
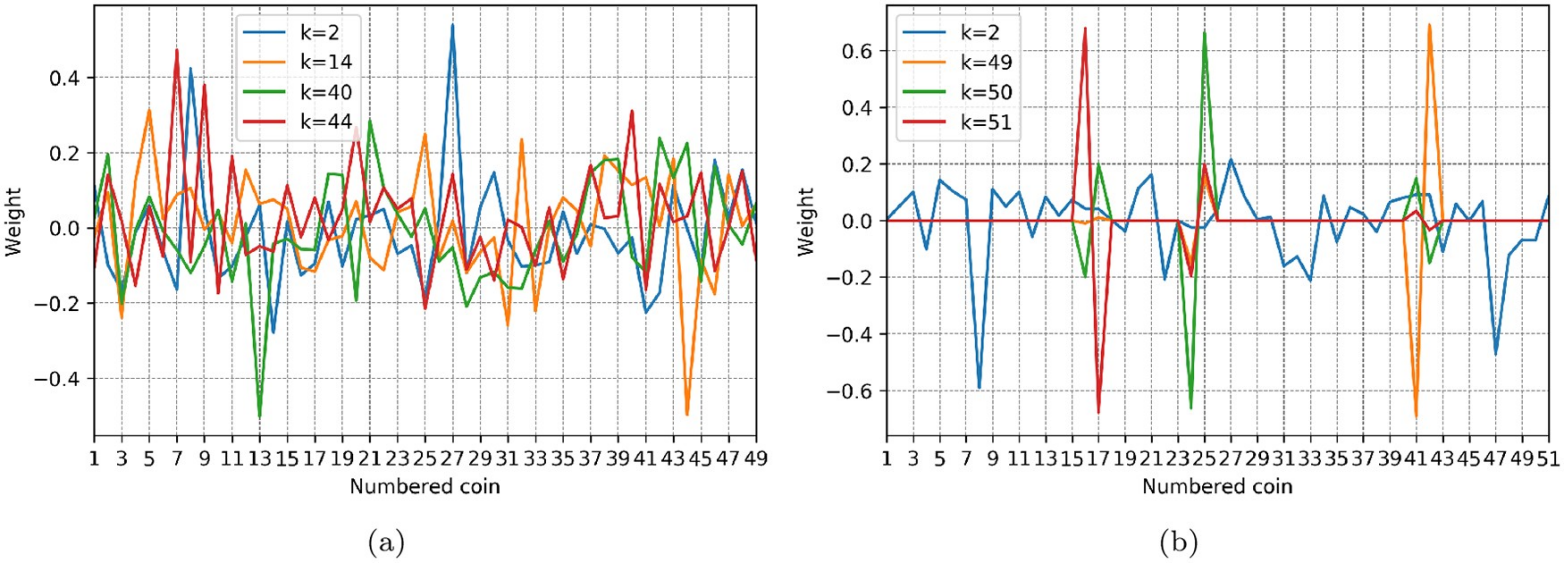
Factor weights. Contribution weight of each coin for the factors with the highest IPR. (a) Eigenvector weights associated with the predictor factors *k* = 2, 14, 40 and 44. (b) Eigenvector weights associated with the response factors *k* = 2, 49, 50 and 51.

Thus, Fig 9 shows a plot of the weight contribution of the predictor and response factors with the highest IPR. Based on a visual inspection, we have chosen the predictor factors identified with *k* = 2, 14, 40 and 44 and the response factors identified with *k* = 2, 49, 50 and 51 as the more localized factors. As a result, returning to the preceding Fig 9, it is observed that there are a few cryptocurrencies with high weights, as expected, while the majority of cryptocurrencies oscillate around zero. This phenomenon is even more representative for the response factors of cryptocurrencies. For example, if we consider cryptocurrencies for which the absolute value of the weight is larger than 0.4, we observe that *tether, steem, bitshares, zilliqa* and *request network* (numbered 7, 8, 13, 27 and 44, respectively) are the more representative cryptocurrencies for the selected predictor factors, whereas cryptocurrencies *iota, zcash, raiblocks, maker, walton, iostoken* and *gxshares* (numbered 8, 16, 17, 24, 25, 41 and 42, respectively) are the equivalent for the response factors (see S1 Table for reference). In general, these factors fluctuate around zero but have a strong peak for a specific currency. These are the corresponding relevant currencies in a predictive model and has to do with the more and less diversified portfolio, and its association with the risk of investing in a portfolio. Specifically, we could explore investing in a mixed portfolio for a predictive model. It can be said that they are the components with the greatest weight that are involved in the predictive model. A model could be tested only with those components as future work.

**Fig 10:**
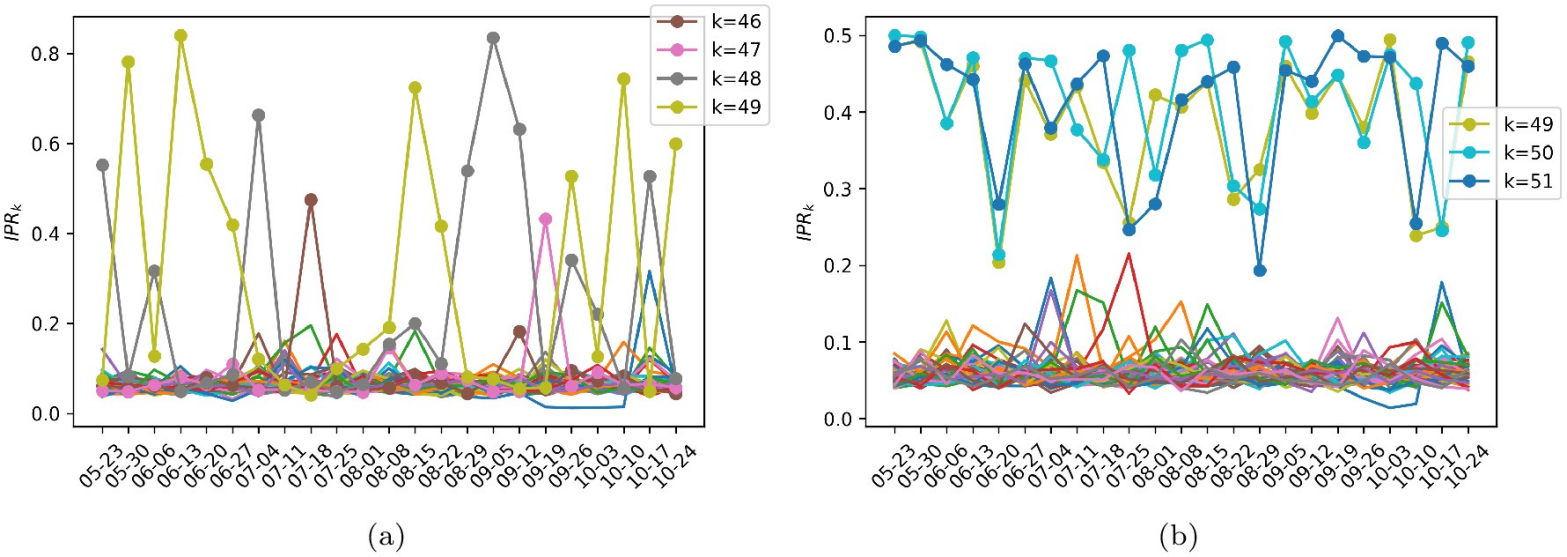
IPR as a function of time. Dynamic inverse participation ratio for the set of subsample data matrices described above. The regularization parameter and level of significance are set to λ = 0.01 and *α* = 0.01, respectively. (a) Predictor factors. (b) Response factors.

Additionally, Fig 10 shows a plot of the dynamic behavior of IPR for each *k* = 1, …, *r*, denoted by *IPR*_*k*_, as a function of time using the same set of subsample data matrices constructed above, where again *r* = *p* for the predictor factors and *r* = *q* for the response factors, and settings *α* = 0.01 and λ = 0.01 are used. It is observed in general that the last factors are more *localized* in the sense of IPR. For example, the last predictor factors 46, 47, 48 and 49 show the highest IPR values consistently over time (see Fig 10a), while the last response factors 49, 50 and 51 exhibit this behavior as well (see Fig 10b). Based on these results, we can call these last factors the *specific-predictor* and *specific-response* modes, respectively.

**Fig 11:**
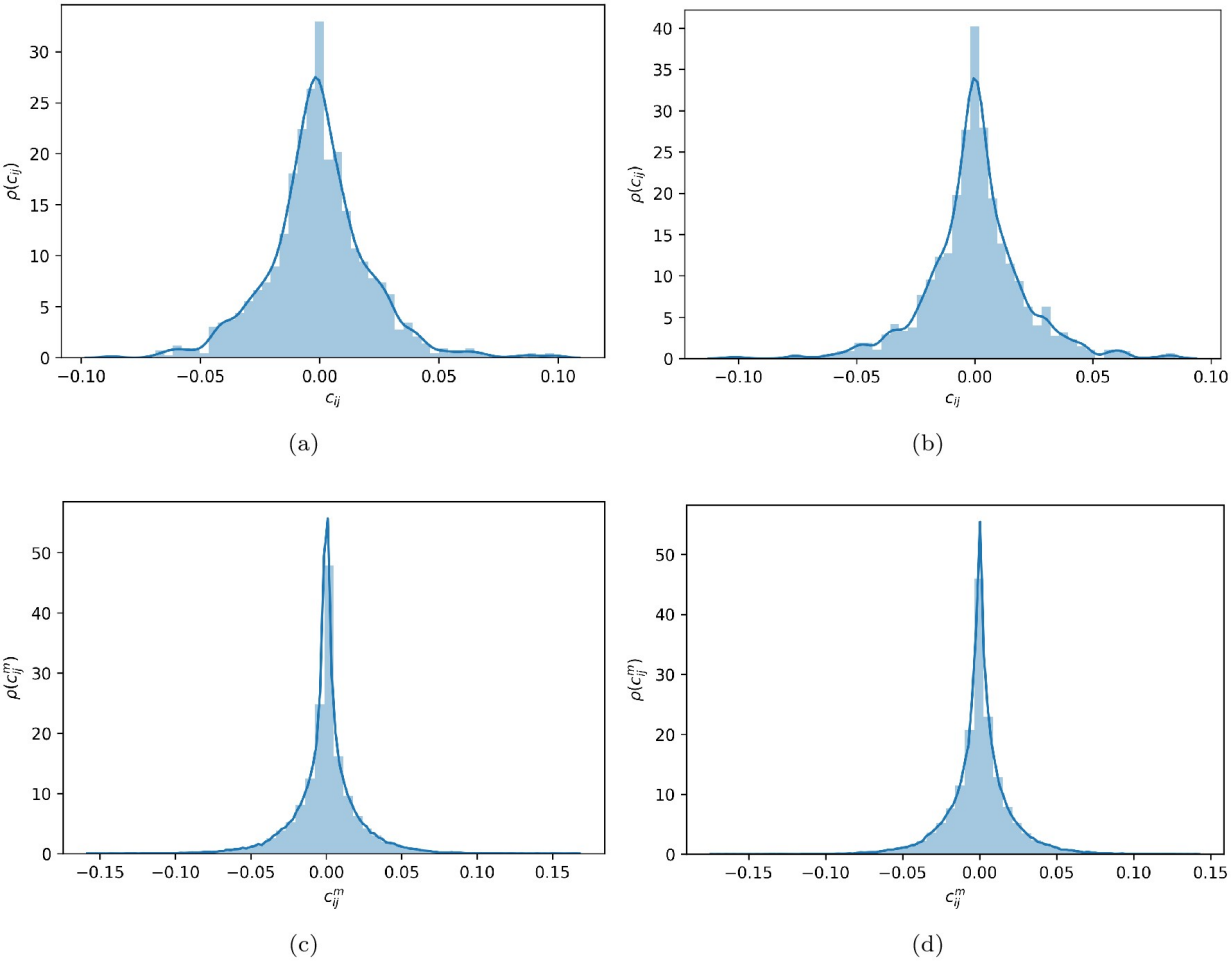
Distribution of correlations between factors. (a) Correlations between predictor factors determined by considering the entire sample period. (b) Correlations between response factors determined by considering the entire sample period. (c) Correlations between predictor factors determined by considering all the subsample components. (d) Correlations between response factors determined by considering all the subsample components. In all cases, settings *α* = 0.01 and λ = 0.01 have been used.

To finalize this analysis, it is important to verify if the factors are correlated in the estimated sample because highly correlated predictor factors provide redundant information about the response and can lead to overfitting. Therefore, Fig 11a and 11b show, for the entire sample period, the normalized distribution *ρ*(*σ*_*ij*_) of correlations between all pairs of factors *σ*_*i*,*j*_, *i*, *j* = 1, …, *r*, where *i* ≠ *j*, and *r* = *p* for the predictor factors, and *r* = *q* for the response factors, respectively. Similarly, Fig 11c and 11d show, for the set of subsample data matrices, the normalized distribution $\rho (\sigma_{ij}^{m})$ of correlations between all pairs of factors $\sigma_{i,j}^{m},i,j=1,\ldots ,r$ and *m* = 1, …, 23, where *i* ≠ *j*, and again, *r* = *p* for the predictor factors, and *r* = *q* for the response factors, respectively. In this last case, symbol *m* represents that the normalized distributions *ρ*(*σ*^*m*^) of correlations *σ*^*m*^ have taken into account *m* = 23 subsample matrices (constructed using the entire six-month sample) to calculate the densities. In all shown cases, settings *α* = 0.01 and λ = 0.01 have been used. In general, Fig 11 does not show significant correlations in any case. Hence, the estimated predictor and response factors are correlated in neither the estimation sample nor the considered subsample windows. Therefore, the predictor factors do not provide redundant information about the response in the predictive model, which out-of-sample analysis does not exploit here because it falls outside the scope of this study and is left for a future study, where different **Γ** structures will be considered (see Eq 21).

## Conclusions

In general, random matrices seem to be a promising tool for performing factor determination in financial and economic problems. Nevertheless, extensive theoretical studies related to random matrices have not yet been applied by practitioners. In part, this lack of use is due to the absence of a simple statistical test that provides reliable results. Accordingly, to fill this gap, we describe the connection between the RRR models and the Tracy-Widom test used to determine the number of significant factors or components in the reduced CCA case of the general RRR models. The main advantage of the proposed procedure is avoiding the subjective element of visual inspection and the computational cost of the resampling approach. Furthermore, the distributional Tracy-Widom test is conceptually related to a more general mathematical framework and involves many branches of basic mathematics and theoretical physics.

In particular, the results obtained here show that a large number of factors are statistically significant in the dataset of cryptocurrencies. Hence, contrary to what a visual inspection suggests, this methodology allows for the retention of more factors. Moreover, the dynamic behavior of the number of factors seems to be related with the dynamic of the composite index of predictor and response cryptocurrencies. As such, future research in this direction could yield insights allowing one to anticipate drops in exchange rates. Furthermore, an IPR analysis suggests the existence of specific-predictor and specific-response factors. These factors are the eigenvectors associated with the smallest singular values obtained using CCA, where only a few cryptocurrencies have remarkable weights and account for the dynamic.

Another contribution of this study is the variable selection methodology based on information theory. We used TE to measure the flow of information between cryptocurrencies’ return variables and specify the predictor and response variables. Since TE can be regarded as a generalization of the Granger causality test under some circumstances, we can cover many scenarios, including possible nonlinear dependencies between the variables. Actually, the STE estimation shows better performance over Granger causality test for simulated systems with linear and nonlinear interactions. The STE can detect the cluster structure, while Granger causality fails to discriminate between the modeled interaction blocks and indicate a higher number of spurious causal dependencies.

In the application to cryptocurrencies we propose a heuristic criterion related to the in-degree and out-degree of the nodes observed if the TE estimation is treated as a graph. Again, the symbolic approach to estimating TE has the advantage of having a distributional test. Therefore, our selected set of response and predictor variables has an associated *p-value*, which is always preferred in the econometric community and makes our results more robust from the statistical perspective. In addition, implementation through the symbolic version is less expensive computationally compared to other approaches such as binning and kernels, and consequently can be used in a large dimensional data set.

The modularity and centrality results give us an understanding of the structure of cryptocurrencies that do not emerge at first glance. We have confirmed that our selected response and predictor variables have a natural cluster structure at least under the modularity approach. Additionally, we have found that modularity validates our selection rule of the sets of predictive and predicted variables. It has also been found that cryptocurrencies called tokens are central in the sense of sending information, while coins are central in the sense of receiving information, in addition to being the authorities under the HITS algorithm. This gives us glimpses of new structures that had not been reported before in the literature and provides a better understanding of the characteristics of cryptocurrencies beyond their predictive power.

An interesting future research direction is to explore other RRR models with a different **Γ** structure and explore the consequences of their predictive power in different scenarios. Similarly, it is exciting to explore further the clustering structure of the cryptocurrencies under the multivariate STE. In addition, its consequences in the theory of portfolios using the significative predicted and predictor eigenvalues to minimize the risk associated with an investment scenario. Finally, it is important to study the case of **Σ ≠ I** to take into account spatio-temporal correlations. The last problem is related to the well-known dynamic factor models in the econometric literature and has the advantage of being more explanatory and linked with structural predictive models such as vector autoregressive (VAR) and vector error correction models (VECM).

## Supporting information

S1 File Raw data. Prices of cryptocurrencies used in this study as described in the Data section before preprocessing.
(XLSX)

S1 Table List of cryptocurrency names. Cryptocurrencies are listed in the descending order of capitalization as of February 2018.
(PDF)

S2 Table Entire list of predictor-response variables. Each set is ordered from the highest to the lowest capitalization of included cryptocurrencies.
(PDF)
