# Supplementary material for: Transfer entropy as a variable selection methodology of cryptocurrencies in the framework of a high dimensional predictive model
Source: PLoS One. 2020 Jan 2;15(1):e0227269. doi: 10.1371/journal.pone.0227269 (PMC6939941; doi:10.1371/journal.pone.0227269)
Supplement: S1 Table — Cryptocurrencies are listed in the descending order of capitalization as of February 2018. (PDF) [file pone.0227269.s002.pdf]

Number	Name	Symbol	Number	Name	Symbol
1	bitcoin	BTC	51	revain	R
2	ethereum	ETH	52	electroneum	ETN
3	ripple	XRP	53	digixdao	DGD
4	Bitcoin cash	BCH	54	gas	GAS
5	litecoin	LTC	55	byteball	GBYTE
6	cardano	ADA	56	Basic attention token	BAT
7	neo	NEO	57	dragonchain	DRGN
8	stellar	XLM	58	digibyte	DGB
9	eos	EOS	59	loopring	LRC
10	iota	IOT	60	Golem network tokens	GNT
11	dash	DASH	61	zilliqa	ZIL
12	nem	XEM	62	bytom	BTM
13	monero	XMR	63	Kyber network	KNC
14	lisk	LSK	64	monacoin	MONA
15	Ethereum classic	ETC	65	pivx	PIVX
16	tron	TRX	66	syscoin	SYS
17	vechain	VET	67	aelf	ELF
18	qtum	QTUM	68	dentacoin	DCN
19	Bitcoin gold	BTG	69	qash	QASH
20	tether	USDT	70	bitcore	BTX
21	icon	ICX	71	cryptonex	CNX
22	omisego	OMG	72	Nebulas token	NAS
23	zcash	ZEC	73	ethos	ETHOS
24	raiblocks	NANO	74	pillar	PLR
25	Binance coin	BNB	75	Power ledger	POWR
26	steem	STEEM	76	iostoken	IOST
27	populous	PPT	77	gxshares	GXS
28	verge	XVG	78	factom	FCT
29	Bytecoin bcn	BCN	79	aion	AION
30	stratis	STRAT	80	salt	SALT
31	siacoin	SC	81	dent	DENT
32	rchain	RHOC	82	funfair	FUN
33	dogecoin	DOGE	83	kin	KIN
34	status	SNT	84	nxt	NXT
35	waves	WAVES	85	cindicator	CND
36	bitshares	BTS	86	zcoin	XZC
37	maker	MKR	87	Enigma project	ENG
38	walton	WTC	88	neblio	NEBL
39	0x	ZRX	89	Polymath network	POLY
40	decred	DCR	90	wax	WAX
41	aeternity	AE	91	chainlink	LINK
42	augur	REP	92	reddcoin	RED
43	komodo	KMD	93	maidsafecoin	MAID
44	veritaseum	KORE	94	Request network	REQ
45	hshare	HSR	95	bancor	BNT
46	ucash	UCASH	96	tenx	PAY
47	Kucoin shares	KCS	97	smartcash	SMART
48	ardor	ARDR	98	santiment	SAN
49	zclassic	ZCL	99	particl	PART
50	ark	ARK	100	blocknet	BLOCK
