# Supplementary material for: Transfer entropy as a variable selection methodology of cryptocurrencies in the framework of a high dimensional predictive model
Source: PLoS One. 2020 Jan 2;15(1):e0227269. doi: 10.1371/journal.pone.0227269 (PMC6939941; doi:10.1371/journal.pone.0227269)
Supplement: S2 Table — Each set is ordered from the highest to the lowest capitalization of included cryptocurrencies. (PDF) [file pone.0227269.s003.pdf]

Number	Predictor	Number	Response
1	ethereum	1	bitcoin
2	neo	2	ripple
3	dash	3	Bitcoin cash
4	monero	4	litecoin
5	lisk	5	cardano
6	Bitcoin gold	6	stellar
7	tether	7	eos
8	steem	8	iota
9	populous	9	nem
10	siacoin	10	Ethereum classic
11	rchain	11	tron
12	dogecoin	12	vechain
13	bitshares	13	qtum
14	0x	14	icon
15	augur	15	omisego
16	komodo	16	zcash
17	veritaseum	17	raiblocks
18	ucash	18	Binance coin
19	Kucoin shares	19	verge
20	revain	20	Bytecoin bcn
21	digixdao	21	stratis
22	gas	22	status
23	byteball	23	waves
24	dragonchain	24	maker
25	loopring	25	walton
26	Golem network tokens	26	decred
27	zilliqa	27	aeternity
28	bytom	28	hshare
29	Kyber network	29	ardor
30	pivx	30	zclassic
31	aelf	31	ark
32	dentacoin	32	electroneum
33	cryptonex	33	Basic attention token
34	Nebulas token	34	digibyte
35	ethos	35	monacoin
36	funfair	36	syscoin
37	kin	37	qash
38	nxt	38	bitcore
39	zcoin	39	pillar
40	Enigma project	40	Power ledger
41	neblio	41	iostoken
42	chainlink	42	gxshares
43	maidsafecoin	43	factom
44	Request network	44	aion
45	bancor	45	salt
46	tenx	46	dent
47	santiment	47	cindicator
48	particl	48	Polymath network
49	blocknet	49	wax
		50	reddcoin
		51	smartcash
